# Deriving mobility-lifetime products in halide perovskite films from spectrally and time-resolved photoluminescence

**DOI:** 10.1126/sciadv.adt1171

**Published:** 2025-04-16

**Authors:** Ye Yuan, Genghua Yan, Samah Akel, Uwe Rau, Thomas Kirchartz

**Affiliations:** ^1^ IMD3-Photovoltaik, Forschungszentrum Jülich, 52425 Jülich, Germany.; ^2^ Faculty of Engineering and CENIDE, University of Duisburg-Essen, Carl-Benz-Str. 199, 47057 Duisburg, Germany.

## Abstract

Lead-halide perovskites are semiconductor materials with attractive properties for photovoltaic and other optoelectronic applications. However, determining crucial electronic material parameters, such as charge-carrier mobility and lifetime, is plagued by a wide range of reported values and inconsistencies caused by interpreting and reporting data originating from different measurement techniques. Here, we propose a method for the simultaneous determination of mobility and lifetime using only one technique: transient photoluminescence spectroscopy. By measuring and simulating the decay of the photoluminescence intensity and the redshift of the photoluminescence peak as a function of time after the laser pulse, we extract the mobility, lifetime, and diffusion length of halide perovskite films. With a voltage-dependent steady-state photoluminescence measurement on a cell, we relate the diffusion length to the external voltage and quantify its value at the maximum power point.

## Teaser

Determine the out-of-plane diffusion length in perovskite thin films with transient photoluminescence.

## INTRODUCTION

The mobility-lifetime product of a semiconductor is one of the most decisive properties governing its suitability as a photovoltaic absorber material (*1*–*7*). However, determining mobilities, lifetimes, and the resulting diffusion lengths typically requires different methods that are often inconsistent (*8*) due to the different injection conditions used. Furthermore, especially in the case of mobility measurements, huge differences (*9*) can result from different types of mobilities that are probed, e.g., intra-grain versus inter-grain mobilities or in-plane versus out-of-plane mobilities (*10*–*12*). The reported mobilities of FAPbI_3_ (*13*–*17*) and MAPbI_3_ (*13*, *18*–*30*) films across various literature sources vary from 10^−1^ to 10^3^ cm^2^ V^−1^ s^−1^. In the case of lifetime measurements, inconsistencies can result from strong injection-level dependence of apparent lifetimes in situations where the decay dynamics are power law rather than exponential (*31*, *32*) as well as capacitive effects in devices that can be misinterpreted as recombination lifetimes (*33*–*37*). Thus, it would be desirable to develop a method that allows measuring both mobility and lifetime with the same approach and enables the determination of lifetimes as a function of injection conditions. Options to do just that are now the photo-Hall method (*30*, *38*, *39*) and the time-resolved photoconductivity measurement (*40*). The photo-Hall method has the caveat that the mobility measured is an in-plane mobility and, hence, is likely more affected by inter-grain transport as the out-of-plane transport required to ensure a high efficiency of a solar cell. Here, we show that the abundantly used transient photoluminescence (PL) also has the potential to determine mobility (μ) and lifetime (τ) if spectral resolution is obtained as a function of time. PL essentially tracks the product of the electron and hole densities as a function of time. As recombination reduces both the electron and hole density in a semiconductor, PL has always been one of the most essential methods to study recombination in lead-halide perovskites (*18*, *41*–*46*) or other semiconductors with sufficient luminescence emission (*47*–*55*). In recent years, the rapidly advancing field of perovskite solar cells and light-emitting diodes has garnered substantial research interest and investment, leading to increasing utilization of PL techniques. These techniques include steady-state PL (*56*, *57*), time-resolved PL (tr-PL) (*18*, *58*), voltage-dependent PL (*59*), temperature-dependent PL (*60*, *61*), and fluence-dependent PL (*56*, *62*). Quantifying charge-carrier transport with PL is comparatively more difficult. Because the initial photogenerated charge carrier density inside the film is a function of depth due to Lambert-Beer–type effects and because lead-halide perovskites are generally lowly doped (*63*), diffusion of electrons and holes will homogenize the carrier profile and, thereby, lead to a reduction in PL intensity (*64*) as well as a redshift of the PL peak due to an increase in reabsorption. This increase in reabsorption is caused by the average depth of emission moving from the front toward the middle of the device. The phenomenon of reabsorption transforms this spatial dependence of emission sites within the emitting perovskite film into a spectral dependence of the emission, a concept that has previously been exploited for instance in Si solar cells (*65*, *66*). In the context of halide perovskites, this phenomenon is pronounced and well visible in thick single crystals (*67*). The effect on films is, however, rather tiny and more difficult to notice and quantify. Thus, it has only recently been used (*10*) to quantify out-of-plane diffusion coefficients in different halide perovskite films.

Here, we combine the quantification of peak shifts with our recently developed high dynamic range transient PL spectroscopy method on the basis of the use of intensified gated charge-coupled device (CCD) cameras, where several measurements using different gain settings are superimposed. We have recently shown that the differential decay times resulting from tr-PL measurements performed at different initial fluences differ in their early time decay but converge to a fluence-independent but carrier-concentration–dependent decay time at later times. Thus, the decay time is a unique function of carrier densities, as expected for recombination of a spatially homogeneous distribution of electrons and holes, only at longer times, while, at earlier times, it retains a memory of the laser fluence. Here, we show that the regions where decay times retain the information on the initial fluence are identical to the parts of the decay where a peak shift in the PL spectrum is visible. Thus, these regions are dominated by changes in the number of reabsorbed photons and are, therefore, indicative of the times, when the electron and hole concentrations are still a function of depth within the film. Consequently, we can distinguish the part of the decay that is affected by diffusion from that affected by recombination alone. Through numerical simulations, we derived the mobility-lifetime (μτ) product, enabling the determination of the diffusion length $L_{D}=\sqrt{\frac{k_{B}T}{q}\mu \tau }$, where *k*_B_*T*/*q* is the thermal voltage. In analogy to the lifetime, the diffusion length exhibits an inverse correlation with the charge carrier density. To accurately relate the diffusion length and carrier lifetime to the external voltage and quantify their values at the maximum power point of a solar cell, it is essential to establish a relationship between Fermi-level splitting Δ*E*_F_ and the external bias voltage. This was accomplished through voltage-dependent steady-state PL measurements conducted on a finished solar cell. The same measurement yields another critical parameter: the carrier exchange velocity of the transport layers. To further elucidate the impact of these parameters on device performance, we have derived a closed-form expression for the current-voltage characteristics as a function of diffusion length, lifetime, and exchange velocity in perovskite devices. This expression provides a comprehensive and quantitative relation between the influence of carrier kinetics on the device performance and can be used to estimate the impact of spectroscopic results on films and layer stacks on eventual performance gains or losses in finished devices. Notably, the results demonstrate that the diffusion length and product of the carrier lifetime and exchange velocity collectively determine the charge collection efficiency, which ultimately governs the overall device performance.

## RESULTS

### Quantifying charge carrier transport

There are essentially two options for how to detect both time-dependent and spectral information during a transient PL measurement. Option 1 is to perform two measurements with different sets of filters and then compare the two measurements. This option is easy to implement with traditional time-correlated single-photon counting equipment and has recently been used by Cho *et al.* (*10*). Option 1 could be implemented by doing two measurements in series, but it could also be done simultaneously using a beam splitter and two photodetectors. Option 2 involves using a detector, which directly captures the whole spectrum for each time delay. Here, one could use intensified CCD cameras with a variable gate or streak cameras. For the current study, we use option 2 using a gated intensified CCD camera from Andor. The investigated samples are triple-cation Cs_0.05_FA_0.73_MA_0.22_PbI_2.56_Br_0.44_ perovskite films directly prepared on Corning glasses with a bandgap of 1.63 eV and thickness of 550 nm.

**Fig. 1.**
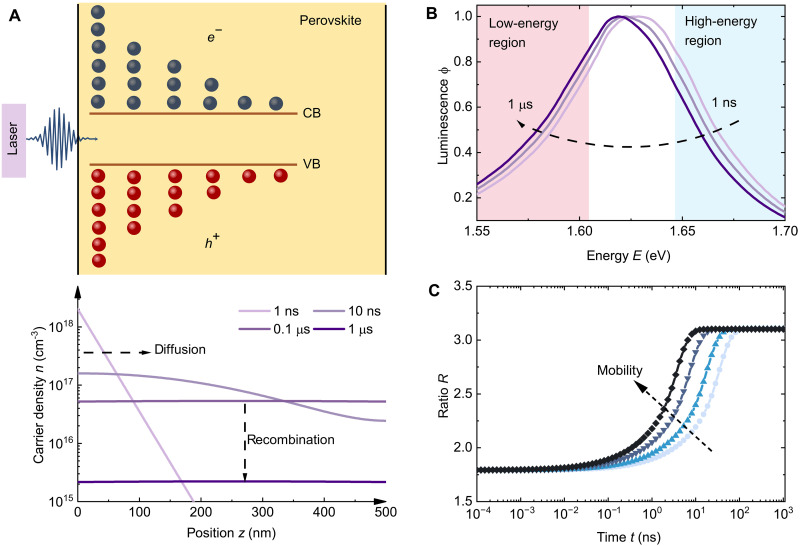
Schematic illustration of carrier diffusion and spectral shift process based on simulated results. (**A**) After the laser pulse hits the sample, the generated carriers in the film will diffuse from one side of the film to the other, while recombination occurs simultaneously. CB, conduction band; VB, valence band. (**B**) Time-dependent PL spectra show an obvious redshift with time, which indicates the reabsorption of PL along with carrier diffusion in perovskite. (**C**) Schematic illustration of the time dependence of the ratio *R*, from which we can quantify the influence of mobility and diffusion coefficients on the spectral shifts. *R* is the ratio of the low-energy region to the high-energy region and defined as $R=\int_{1.45}^{1.58}\phi \mathrm{dE}/\int_{1.68}^{1.80}\phi \mathrm{dE}$. Note that *R*(*t*) may have slightly different shapes than shown in (C) in the presence of, e.g., trapping/detrapping effects (cf. section S2), but it will always show a feature related to the mobility.

A schematic representation of the experiment is shown in Fig. 1. After excitation, photocarriers are generated primarily close to the front side of the perovskite film. The initial carrier distribution (at point *t* ≈ 0) is determined by the absorption coefficient α of the film (section S2) and the wavelength λ of the excitation laser (here, α = 4 × 10^5^ cm^−1^ and λ = 343 nm, respectively). Here, we intentionally use an ultraviolet (UV) laser with a low wavelength where the perovskite has a high absorption coefficient. The lower the wavelength, the higher the absorption coefficient of the perovskite layer at the laser wavelength and the more abrupt the initial carrier profile will be as a function of depth within the film. Driven by the concentration difference, the carriers diffuse from the front toward the back side of the film until the carrier concentration is approximately independent of position. Depending on the recombination velocities at the two surfaces, gradients of the concentrations of electrons and holes toward the surfaces may remain. The time that it takes for the carrier concentrations to homogenize depends on the mobility of electrons and holes. In the example situation presented in Fig. 1A, where we chose a mobility of 1 cm^2^/Vs for electrons and holes, we see that carrier diffusion mainly occurs in the first tens of nanoseconds (as demonstrated in the lower panel figure). After 100 ns, the carrier concentrations are nearly perfectly flat, and, from then, onward recombination is the only driver of a further reduction in both PL and carrier densities. Using a gated CCD setup, we can obtain a series of time-dependent PL spectra, which we later transform into a transient PL decay curve. The transient decay curve provides information about charge-carrier recombination, while the time-dependent PL spectra reflect the process of carrier diffusion. The schematic illustration of the measurement technique is shown in fig. S1. As shown in Fig. 1B, the simulated time-dependent PL spectra corresponding to the carrier profiles seen in Fig. 1A show a redshift with time caused by diffusion and photon reabsorption (*67*). In our measurements, the detector is positioned on the same side as the excitation laser. The PL caused by radiative recombination must pass through the film before being detected and thus being reabsorbed. Reabsorption primarily reduces the luminescence on the high-energy flank of the spectrum, thereby reducing the peak energy. As the charge carriers diffuse deeper into the film, more PL is absorbed. Thus, the spectra exhibit a redshift over time.

There are different ways to quantify the spectral shift. The most intuitive option might be to plot the PL peak as a function of time after excitation. However, for noisy data, the peak itself is a poor indicator of spectral shifts as it relies on one single point of the spectrum (the highest point) and depends on the resolution of the spectrometer. A better option would be to use the whole spectrum for quantifying the shift. This could be achieved, e.g., via calculating the center of mass or via determining the PL intensity ratio of the low-energy region to the high-energy region. We chose the latter approach that was already used previously by (*10*), because it will be more easily applicable to researchers using time-correlated single photon counting (TCSPC) setups for the experiment. In fig. S3, we compare these three methods, demonstrating that the variation of the center of mass contains the same information as that of the ratio, while the change in the peak (of the experimental data) is not suitable as an effective indicator owing to its requirement for high spectral resolution and low noise. Here, we define the spectral shift ratio as $R=\int_{1.45}^{1.58}\phi \mathrm{dE}/\int_{1.68}^{1.80}\phi \mathrm{dE}$. The exact integration boundaries will always be somewhat arbitrary and must be adjusted to the spectral position of the PL spectrum. Thus, the absolute value of *R* will be largely irrelevant and strongly dependent on the integration boundaries. However, the relative change in *R* as a function of the time delay after the laser pulse will be decisive in quantifying the mobility. When the mobility increases, the ratio increases rapidly until it reaches a plateau (Fig. 1C), which indicates the equilibrium stage of the carrier distribution. In section S2, we use the same model (i.e., assuming the SRH recombination is dominated by deep traps in the bulk and surfaces, which is close to the case of traditional semiconductors), and the influence of other factors was investigated. Specifically, the absorption coefficient affects the initial value of the ratio, while the film thickness and surface recombination velocity affect the plateau value. We further investigated the influence of shallow defects, as shown in section S3. The results demonstrate that the detrapping effect of shallow defects markedly alters the shape of the plateau observed in the schematic in Fig. 1C at longer times. Thus, in the presence of shallow traps, the ratio *R* may not be saturating at all but is continuously changing, which slightly complicates the assignment of a mobility to the abrupt change in the ratio *R*.

### PL experiments

**Fig. 2.**
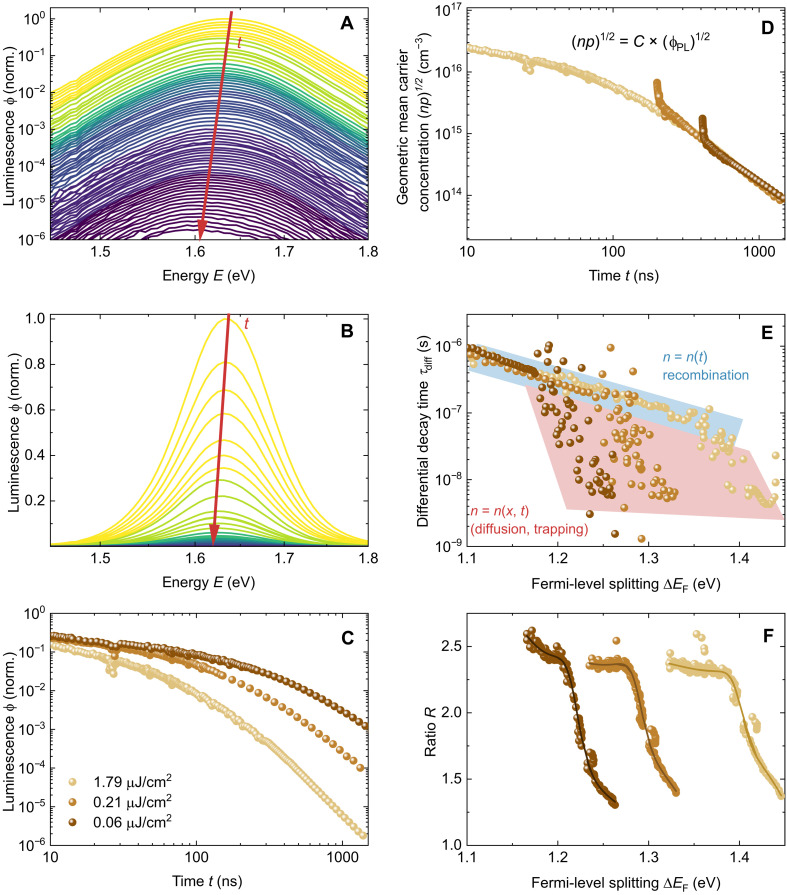
Tr-PL decay and spectral shift of experimental data for different illumination intensities. (**A**) PL spectra at different time delays during the measurement with an illumination intensity of 1.79 μJ/cm^2^ using a logarithmic scale. (**B**) PL spectra at different time delays during the measurement with an illumination intensity of 1.79 μJ/cm^2^ using a linear scale. (**C**) PL decay curves at three different fluences. (**D**) Decay curves of carrier concentration with shifted time axis. The average carrier concentration over volume is used at the initial time (*t* ≈ 0). (**E**) Differential decay time versus Fermi-level splitting. (**F**) Spectral shift ratio versus Fermi-level splitting. The solid lines in [(E) and (F)] indicate trends in the experimental data.

In Fig. 2, the tr-PL data measured using the gated CCD setup with different fluences are displayed. The recorded time-dependent PL spectra in Fig. 2A exhibit an obvious peak shift. Specifically, the PL spectra at different times are shown in Fig. 2B on a linear scale that is a more frequently seen way to display PL spectra in the literature. Figure 2C presents the tr-PL decay curves, which exhibit a slower decay at lower illumination intensities. In Fig. 2D, the luminescence is transformed into carrier concentration, and the time axis is adjusted to merge the curves. The merged curves show two distinct parts that we will refer to as the partial differential equation (PDE) and the ordinary differential equation (ODE) parts of the decay. The rationale for this naming convention is that the PDE parts of the decay are those where different fluences lead to different decay times at a given spatially averaged carrier density. This is the case, when the carrier densities are functions of time and space and when they are mathematically the result of solving a PDE in time and space. Once the homogenization of *n*(*x*) and *p*(*x*) is approximately complete, the decay times are only a function of carrier densities or Fermi-level splittings but not anymore of fluence. In this case, the whole mathematical problem can be broken down into the solution of an ODE in time. Thus, the PDE part is the part that contains information about transport and recombination, while the ODE part is the part that contains information only about recombination.

Although the ODE parts of the different curves generally overlap, the initial parts of each decay (PDE part) are noticeably outside the ODE part. In Fig. 2E, the Φ_PL_ versus *t* plot (Fig. 2C) is transformed into the τ_diff_ versus Δ*E*_F_ plot based on $\tau_{\text{diff}}={\left[-\frac{1}{2}d\text{ln}\left(\phi_{\text{PL}}\right)/dt\right]}^{-1}$ [the factor ^1^/_2_ corresponds to high-level injection; (*68*)], $\Delta E_{F}\left(0\right)=k_{B}T\text{ln}\left[n\left(0\right)p\left(0\right)/n_{i}^{2}\right]$ and $\phi_{\text{PL}}\propto \text{exp}\left(\Delta E_{F}/k_{B}T\right)$ (*68*, *69*). Note that we only assume *n* = *p* at *t* ≈ 0 s. At longer times, *n* and *p* are allowed to diverge due to asymmetric trapping and detrapping. For instance, in an intrinsic semiconductor, once the excited electrons are trapped by acceptor-like defects, an excess of free holes is generated in the valence band. This photoinduced asymmetry between electron and hole densities is often referred to as photodoping and has a considerable impact on the charge carrier dynamics in perovskites (*70*), which is included in our model. The initial carrier concentration Δ*n*(0) after the laser pulse is estimated on the basis of the measured laser power densities. Similar to the curves in Fig. 2D, all curves can be divided into PDE and ODE parts. Additionally, the spectral shift ratio *R* acquired from the time-dependent PL spectra is depicted in Fig. 2F. The ratio *R* can be divided into a time-dependent and an approximately time-independent part. At early times, the carriers diffuse from the front of the film into the bulk until they are homogeneously distributed throughout the film. While the diffusion process occurs, the ratio *R* is time dependent. Once the homogenization process is complete, the ratio remains stable. Comparing Fig. 2E and Fig. 2F, we found that the PDE parts of the decay curves correspond to the time-dependent parts of the ratio *R*, indicating that carrier diffusion is one of the primary physical processes involved. The change in carrier concentration is governed by both time and position, expressed as *n* = *n*(*x*, *t*). Conversely, in the ODE component, it is sufficient to track the evolution of the average carrier density *n*_av_ as a function of time, where *n*(*x*) ≈ *n*_av_ and *n*_av_ = *n*_av_(*t*). During the PDE part of the decay, it matters whether a given average carrier density was created 30 or 300 ns ago by the laser pulse because the two cases will show different *n*(*x*) profiles. Thus, the PDE part depends on the fluence, whereas the ODE part depends only on the average carrier density or Fermi-level splitting.

### Inferring the mobility by numerical simulations

**Fig. 3.**
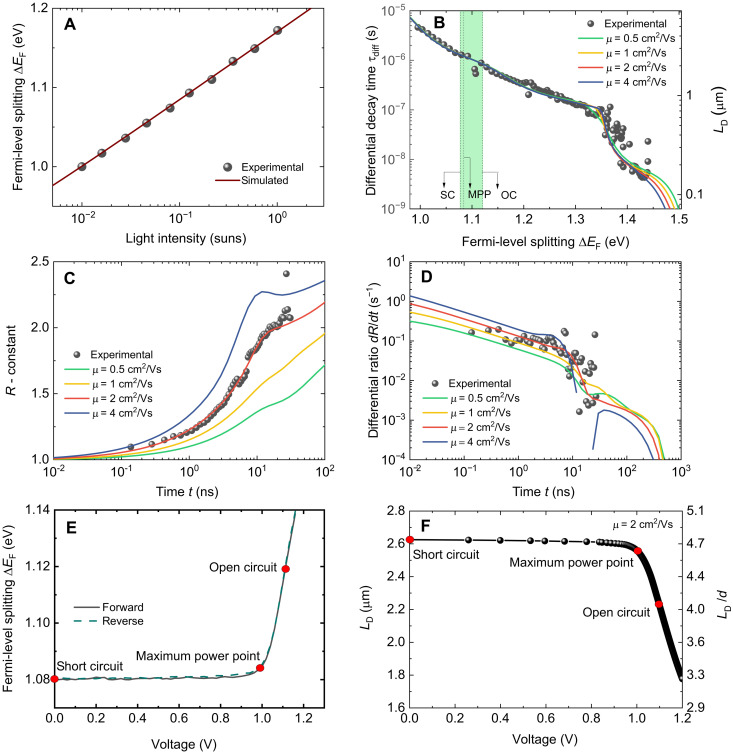
Experimental and simulation results. (**A**) Experimental results of Δ*E*_F_ versus illumination intensity acquired from steady-state PL results and the corresponding simulation results. (**B**) Experimental differential decay time versus Fermi-level splitting acquired from gated CCD setup and the corresponding simulated results with different carrier mobilities. On the *y* axis (right side), we calculated $L_{D}=\sqrt{D\tau_{\text{diff}}}$, where D=μkT/q and μ = 2 cm^2^/Vs. SC, short circuit; OC, open circuit; MPP, maximum power point. (**C**) Experimental ratio versus time acquired from gated CCD setup and the corresponding simulated results with different carrier mobilities. Here, we shift the *R* by subtracting a constant to obtain the same starting value. (**D**) The corresponding differential ratio value versus time from the experiment and simulations. (**E**) Experimental results of Δ*E*_F_ versus the external bias voltage acquired from the voltage-dependent PL measurement under 1-sun light intensity. (**F**) The relationship between *L*_D_ and external bias voltage, which is acquired from the reverse scan result of (E) and the simulated result (μ = 2 cm^2^/Vs) of (B). On the *y* axis (right side), we calculated *L*_D_/*d*, where *d* is the thickness of the film.

On the basis of the experimental steady-state and transient PL data, we perform numerical simulations to extract the mobility and differential decay time of our sample. We used two models for the simulation to confirm the reliability of the results. A detailed description of the numerical models can be found in Materials and Methods and section S5. The first is a zero-dimensional (0D) model, which primarily focuses on trapping, detrapping, and recombination. It can simultaneously fit both the steady-state (Fig. 3A) and transient PL experimental data (fig. S5), as reported in our previous work (*32*). However, this model does not consider the spatial dependence of the carrier density as a function of depth within the film. Thus, it cannot be used to simulate the PL peak shift data. Figure 3A shows that the calculated Δ*E*_F_ from steady-state PL data measured under various illumination intensities agrees well with the simulated result. Subsequently, to better understand the carrier dynamics, we used a 1D model, which not only contains the trapping, detrapping, and recombination processes but also incorporates diffusion and reabsorption processes and is thereby able to reproduce and fit the PL peak shift. The simulated results are presented in Fig. 3 (B to D). The values of fitting parameters for trapping, detrapping, and recombination are present in both models and are identical, as listed in table S1. Figure S5 presents the simulated transient PL results (τ_diff_ versus Δ*E*_F_ plot) using these two models. It shows that the two fitted curves are almost overlapping except for the PDE part, which is consistent with the fact that the 0D model does not consider the carrier diffusion process. For the ODE part, both models fit the data well because the 0D model is identical to the 1D model in the limit of a homogeneous carrier distribution. As depicted in Fig. 3B, both the differential decay time $\tau_{\text{diff}}$ and diffusion length $L_{D}=\sqrt{D\tau_{\text{diff}}}$ increase with decreasing Δ*E*_F_. From the simulated results for different mobilities, we observe that the impact of mobility is primarily confined to the PDE parts of decay, while the ODE of the decay remained relatively unchanged. However, the changes brought about by mobility to the PDE part of the decay are tiny, and the precision of the present measurement is not sufficient to accurately determine the mobility. In contrast, the spectral shift ratio and differential ratio are more sensitive to the variation of mobility, as demonstrated in Fig. 3 (C and D). Note that, as tiny changes in bandgap energy (1 meV) would result in large differences in the value of *R* (fig. S7), we suggest that the variation trend of *R* and the value of *dR*/*dt* are more important for analyzing carrier diffusion. Conversely, the absolute value of *R* will be largely irrelevant. Therefore, we shift the *R* by subtracting a constant, as shown in Fig. 3C. The subtracted constants are somewhat arbitrary depending on the situation. The idea for the subtraction (i.e., shifting) is to make the initial position of the experimental data similar to that of the simulation curves, which will not affect the trend of *R* or the value of *dR*/*dt*. Note that we only simulate the first ~10 ns for the redshifted data (diffusion related; Fig. 3, C and D) to avoid the detrapping effect of the shallow traps, which is sensible as the film thickness is only ~500 nm. Nevertheless, we simulate the whole-time region (until ~6000 ns) to extract the recombination-related parameters using the 1D model (Fig. 3B). Consequently, we determined a mobility μ ≈ 2 cm^2^/Vs of our perovskite film sample, as well as a diffusion coefficient *D* ≈ 0.052 cm^2^/s, which was calculated using the Einstein relation $D=\mu k_{B}T/q$, where *k*_B_*T* is the thermal energy and *q* is the elementary charge. For simplicity, we did not distinguish electron and hole mobilities/diffusion coefficients. Note that the sample exhibits similar mobility under lower illumination intensities (fig. S8). Photon recycling is known to effectively slow down radiative recombination, thereby enhancing the open-circuit voltage of perovskite solar cells (*71*). Furthermore, photon recycling can accelerate the homogenization of electron and hole densities and, thereby, the shift of the PL spectra. This can potentially lead to an overestimation of the electronic transport parameters μ and *D* (*67*). In our case, we estimate this influence to be minimal, as discussed in section S1. Figure 3E presents the relationship between Δ*E*_F_ and the external bias voltage, which is obtained from the voltage-dependent PL measurement performed under 1-sun illumination condition. Moreover, the diffusion length as a function of the external bias voltage is displayed in Fig. 3F, which is determined by combining the results shown in Fig. 3 (B and E). We observe that the sample exhibits an *L*_D_ of ~2.6 μm at short-circuit condition and maximum power point and ~2.2 μm at open-circuit condition.

### Physical processes underlying the τ_diff_ versus Δ*E*_F_ plot

Three essential processes affect the carrier concentration within a perovskite film after the excitation by a laser pulse: (i) the diffusion of carriers, (ii) the trapping of free carriers by shallow defects, and (iii) the transition of trapped carriers to the valence band. These processes correspond to distinct regions in the τ_diff_ versus Δ*E*_F_ plot. In the following, we will elaborate on these processes assuming for simplicity that the shallow traps are closed to the conduction band. For shallow traps close to the valence band, every instance of the word “electron” would have to be replaced by “hole” and vice versa.

**Fig. 4.**
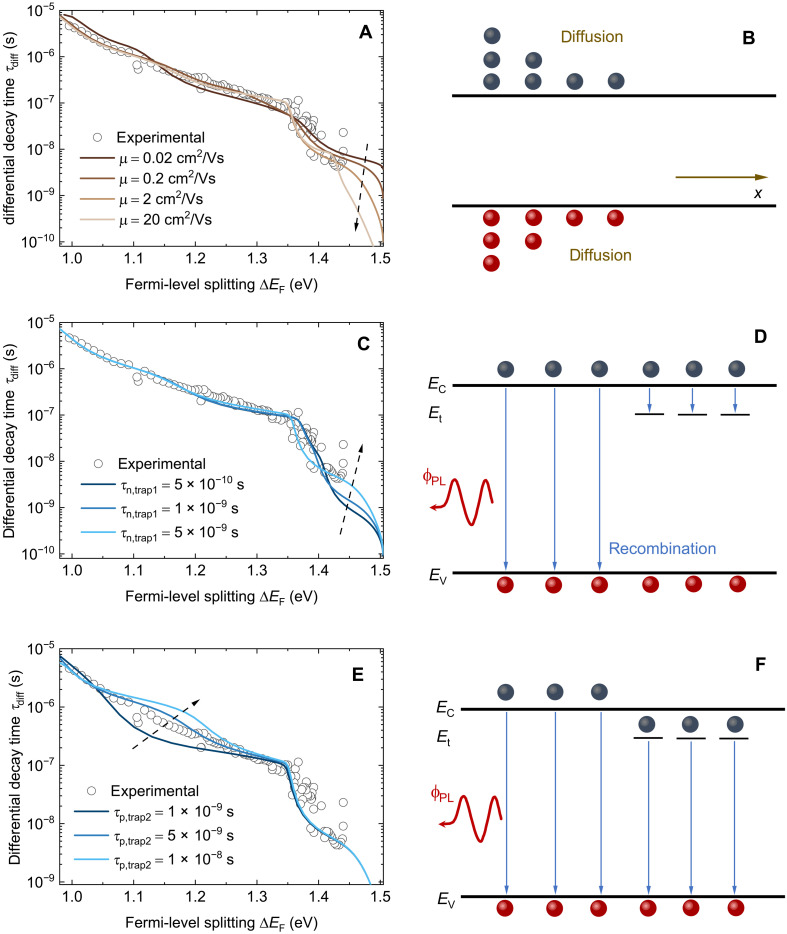
Analysis of factors influencing τ_diff_ versus Δ*E*_F_ plot and corresponding physical processes. (**A** and **B**) Influence of carrier diffusion on the onset value of τ_diff_. The carrier mobility was adjusted. (**C** and **D**) Influence of electron trapping process on the PDE part of τ_diff_. The electron lifetime related to trap 1 is adjusted by changing the electron capture coefficient $\beta_{n}$ while maintaining a constant trap density $N_{t}$. (**E** and **F**) Influence of the hole capture process on the ODE part of τ_diff_. The hole lifetime related to trap 2 was adjusted by changing the hole capture coefficient $\beta_{p}$ while maintaining a constant trap density $N_{t}$. We further show the influence of the hole lifetimes related to trap 1 and trap 3 in figs. S11 and S12. *E*_c_, energy level of conduction band edge; *E*_t_, energy level of trap; *E*_v_, energy level of valence band edge.

Immediately after excitation, the photogenerated carriers begin to diffuse from the illuminated side to the other side. As demonstrated in Fig. 4A, carrier diffusion has a substantial impact on the onset value of τ_diff_, which, in turn, affects the shape of the curve in the high Fermi-level region (1.43 to 1.5 eV). Additionally, trapping by defects is another important physical process that may affect the PL decay at early times. Similar to the diffusion process, it also occurs right after the excitation. In Fig. 4C, we adjust the electron capture coefficient $\beta_{n}$ of the shallowest trap while maintaining a constant trap density $N_{t}$. Consequently, the electron lifetime $\tau_{n}$ is altered accordingly and is given by $\tau_{n}=1/\left(N_{t}\beta_{n}\right)$. As shown in Fig. 4C, the electron lifetime plays a crucial role in shaping the PDE part. For a shorter electron lifetime, which implies more effective electron capture by the defect, the Δ*E*_F_ shows a faster decrease and the trapping process is shortened. The impact of the trapping process is primarily observed in the higher Δ*E*_F_ range (e.g., 1.4 to 1.5 eV) and at early times (fig. S9B). Furthermore, Fig. 4C reveals that, although the carrier trapping process affects the shape of the PDE part, it has little influence on the onset point. In fig. S10, a simpler scenario where only one shallow trap and a low radiative recombination coefficient are used further demonstrates the impact of trapping on the PDE part, which supports the aforementioned argument. The final stage of the carrier transport process involves the non-radiative recombination of trapped electrons with holes in the valence band. This process is primarily dependent on the hole lifetimes, which determines the shape of the ODE part. The hole lifetime is calculated as $\tau_{p}=1/\left(N_{t}\beta_{p}\right)$, where $\beta_{p}$ is the hole capture coefficient. As shown in Fig. 4E, we adjusted the hole lifetime $\tau_{p}$ for trap 2 (the middle one) and observed that a lower hole lifetime leads to a smaller τ_diff_ for a given Δ*E*_F_. Because we included three traps in the simulation, they affect different regions of Δ*E*_F_ sequentially. From shallow to deep, the traps influence the high, middle, and low Δ*E*_F_ regions, corresponding to early, middle, and late time regions, respectively (see figs. S9, S11, and S12).

### Numerical simulation of device performance

In the following, we want to put the obtained parameters into perspective. This can be done using numerical simulations or using analytical approximations. For the sake of achieving a more intuitive approach to the influence of absorber layer diffusion and transport layer mobility on charge collection, we derived an approximative but analytical equation for the current-voltage curve of a perovskite solar cell. Similar equations have been derived to describe the solar cell performance for doped semiconductors [e.g., doped silicon (*72*) and Cu(In,Ga)Se_2_ (*73*)] and very thin intrinsic semiconductors [e.g., amorphous silicon (*74*)]. However, for the case of perovskites, which are intrinsic semiconductors with a high permittivity absorber and are combined with low permittivity, low conductivity transport layers, so far, no closed-form expressions for the current-voltage curve as a function of the diffusion lengths and extraction speed through the transport layers have been published. This equation effectively includes the influence of ions by assuming a perfectly field-free absorber layer, where the field is screened by a sufficiently high ion density. Furthermore, it includes bulk recombination and diffusion within the perovskite and considers transport through the electron- and hole-transport layers by an effective exchange velocity *S*_exc_. This exchange velocity can be easily determined from voltage-dependent PL measurements (*35*), whereas the diffusion length and bulk recombination lifetime can be deduced from the tr-PL measurements, as shown in Fig. 3. We can then write the current-voltage curve as (see section S4 for detailed derivation)

$$J=\mathrm{qd}\left[\frac{\frac{L_{D}}{d}\text{tanh}\left(\frac{d}{L_{D}}\right)}{\frac{L_{D}}{\tau_{\text{diff}}S_{\text{exc}}}\text{tanh}\left(\frac{d}{L_{D}}\right)+1}\right]\left\{\frac{n_{0}}{\tau_{\text{diff}}}\left[\text{exp}\left(\frac{qV_{\text{ext}}}{2k_{B}T}\right)-1\right]-G\right\}$$ (1)

where *n*_0_ is the intrinsic carrier concentration, *V*_ext_ is the external voltage, *G* is the average generation rate throughout the perovskite layer, and *S*_exc_ is the carrier extraction velocity of the transport layer. Our derivation of Eq. 1 is based on earlier work primarily by Sandberg *et al*. (*75*) and Rau *et al.* (*73*) and provides a more intuitive understanding of extraction losses as compared to purely numerical simulation results. Note that Eq. 1 contains recombination and transport as parameters. However, as our experimental assay of recombination originates from a film, the resulting current-voltage (*J*-*V*) curve will provide a hypothetical *J*-*V* curve in the absence of interfacial recombination. Thus, the equation predicts a higher open-circuit voltage *V*_oc_ than that of the actual device, whereby the difference is due to interfacial recombination losses that are absent from the lifetime data obtained on films.

The prefactor $\left[L_{D}\text{tanh}\left(d/L_{\text{diff}}\right)/d\right]/\left[1+L_{D}\text{tanh}\left(d/L_{D}\right)/\left(\tau_{\text{diff}}S_{\text{exc}}\right)\right]$ in Eq. 1 can be intuitively understood as a collection efficiency, which is related to *L*_D_ and *S*_exc_. Note that *L*_D_ and *S*_exc_ represent properties of the perovskite absorber and transport layers, respectively. The parameter *S*_exc_ describes how fast the transport of electrons through the electron transport layer (ETL) and holes through the hole transport layer (HTL) is, and it can be described as $S_{\text{exc}}=\frac{\mu_{\text{CTL}}U_{\text{CTL}}/d_{\text{CTL}}}{1-e^{\left[-\left(U_{\text{CTL}}/kT\right)\right]}}$, where μ_CTL_ is the mobility of the charge-transport layer (CTL), *U*_CTL_ is the potential difference across the CTL, and *d*_CTL_ is the CTL thickness (*35*, *76*). At steady state, the *S*_exc_ can be extracted from the voltage-dependent PL measurements via (*35*)

$$S_{\text{exc}}=J/\left\{qn_{0}\left[\text{exp}\left(\frac{qV_{\text{ext}}}{2k_{B}T}\right)-\text{exp}\left(\frac{{\mathrm{qV}}_{\text{int}}}{2k_{B}T}\right)\right]\right\}$$ (2)

**Fig. 5.**
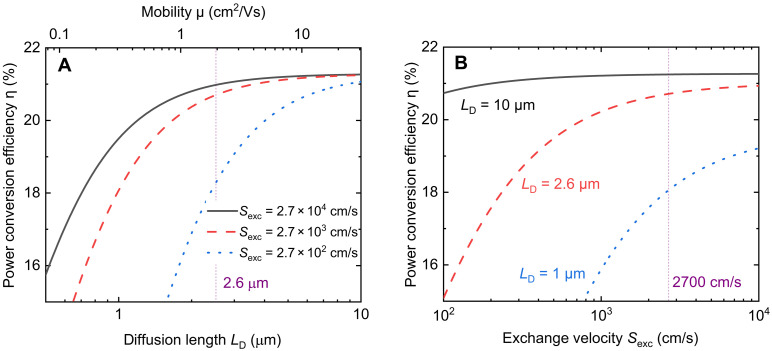
Numerical simulation of device performance. The simulated power conversion efficiency as a function of (**A**) diffusion length *L*_D_ and (**B**) exchange velocity *S*_exc_ using Eq. 1. The mobility μ in (A) was calculated using $L_{D}=\sqrt{\frac{k_{B}T}{q}{\mu \tau }_{\text{diff}}}$ by assuming τ_diff_ = 1.3 μs. The purple dashed lines in the figures at (A) *L*_D_ = 2.6 μm and (B) *S*_exc_ = 2700 cm/s indicate the approximate experimental values of our sample.

For our device, the *S*_exc_ is around 2700 cm/s within the range of voltages relevant for 1-sun operation (fig. S14). Other parameters for the calculation are shown in fig. S15 and table S2. Figure 5 (A and B) exhibits the variation of power conversion efficiency η along with diffusion length *L*_D_ and exchange velocity *S*_exc_, respectively. The efficiency was simulated by using Eq. 1. As shown in Fig. 5A, with increasing *L*_D_, the efficiency η increases at the beginning, but, for higher values of *L*_D_, the efficiency increase slows down until it reaches a plateau. It can be observed that, when the absorber mobility increased from 1 to 10 cm^2^/Vs (i.e., *L*_D_ increased from 1.8 to 5.7 μm), the efficiency of the sample with *S*_exc_ = 2700 cm/s (the red curve) can increase by 4.9%. However, when the mobility drops by an order of magnitude (from 1 to 0.1 cm^2^/Vs, i.e., *L*_D_ decreased from 1.8 to 0.57 μm), the efficiency markedly decreased by 31%. Moreover, the decline would be more severe for the sample with a slower *S*_exc_ (blue curve). The situation of the effect of *S*_exc_ on efficiency is similar. When the *S*_exc_ is sufficiently fast, the increasing trend of the efficiency slows down until reaching a plateau. For a certain *L*_D_, for instance, *L*_D_ = 2.6 μm (the red curve in Fig. 5B), the efficiency increases by 5.1% when the *S*_exc_ increases from 10^3^ to 10^4^ cm/s, while it markedly decreases by 25% when the *S*_exc_ drops from 10^3^ to 10^2^ cm/s.

Regarding Eq. 1, in the case of *L*_D_ >> *d*, we can make the approximation tanh(*d*/*L*_D_) = *d*/*L*_D_ by using the Taylor expansion, and, hence, the collection efficiency can be simplified to ${\left[d/\left(\tau_{\text{diff}}S_{\text{exc}}\right)+1\right]}^{-1}$. Therefore, the current-voltage curve can be written as (*35*)

$$J=\mathrm{qd}\left(\frac{1}{\frac{d}{\tau_{\text{diff}}S_{\text{exc}}}+1}\right)\left\{\frac{n_{0}}{\tau_{\text{diff}}}\left[\text{exp}\left(\frac{qV_{\text{ext}}}{2k_{B}T}\right)-1\right]-G\right\}$$ (3)

The *L*_D_ cancels out such that the result is independent of the bulk mobility, which explains the phenomenon of the plateau for sufficiently long *L*_D_. In our case, the ratio *d*/*L*_D_ ≈ 0.2, which satisfies the approximation of tanh(*d*/*L*_D_) = *d*/*L*_D_ (see fig. S16), suggesting a collection efficiency of ${\left[d/\left(\tau_{\text{diff}}S_{\text{exc}}\right)+1\right]}^{-1}$. Furthermore, in the case of $\tau_{\text{diff}}S_{\text{exc}}\gg d$, the collection efficiency approaches 1 (which means sufficiently efficient charge carrier extraction); therefore, further increasing *S*_exc_ would not have a substantial impact on the efficiency. In contrast, if *S*_exc_ is getting smaller, then the denominator of the collection efficiency will rapidly increase, which explains the marked decrease in the efficiency with decreasing *S*_exc_. In our case, the ratios *d*/*L*_D_ ≈ 0.2 and τ_diff_*S*_exc_/*d* ≈ 6800 approximately meet the conditions of *L*_D_ >> *d* and τ_diff_*S*_exc_ >> *d*, thereby ensuring good device performance (see fig. S17). Therefore, while a gain in any of the parameters would lead to small gains in efficiency, a drop in *L*_D_ and *S*_exc_ would have a substantial negative impact on the efficiency.

## DISCUSSION

In this study, we investigated the charge-carrier dynamics of perovskite thin films through a detailed analysis of spectrally and tr-PL data. The data collected on both PL decay and PL redshift over time shed light on the carrier recombination and diffusion processes. Our findings indicate that the onset of tr-PL decay is determined by the electron lifetime with the underlying physical process being the electron capture by shallow defects. After excitation, the carriers accumulate on the excitation side, leading to carrier diffusion due to concentration differences. Two distinct parts appear in the PL decay. The initial part, named PDE part, appears during the stage, where the time-dependent PL spectra exhibit a substantial spectral redshift. This redshift occurs due to photon reabsorption and gradually disappears when carriers are evenly distributed with mobility playing a dominant role in this process. Mathematically, this part is the solution of a PDE in space (depth within the film) and time. The following part of the decay curves is determined entirely by recombination and is not dependent on charge-carrier transport anymore. Mathematically, this part of the decay follows from the solution of an ODE in time and is consequently termed ODE part.

Through simulations of the PL decay and PL redshift results, we determined parameters for both carrier recombination and diffusion. Our findings suggest that the mobility and diffusion coefficient of the perovskite thin film are ~2 cm^2^/Vs and 0.052 cm^2^/s, respectively. Additionally, we found that the lifetime increases with a lower Δ*E*_F_ value, resulting in an anticorrelation between mobility-lifetime products and injection level. Note that we assumed acceptor-like defects close to the conduction band in our discussion. If the traps are donor-like defects close to the conduction band, then the electron-hole types mentioned above would be swapped. This study provides a comprehensive understanding of the carrier kinetics of perovskite thin films and presents a unified method for measuring carrier mobility and lifetime.

## MATERIALS AND METHODS

### Materials

All chemicals were used as received, and the details are as follows: methylammonium iodide (MAI; Greatcell Solar), formamidinium iodide (FAI; Greatcell Solar), cesium iodide (CsI; 99.9%; Alfa Aesar), lead(II) iodide (PbI_2_; 99.99%; TCI), lead bromide (PbBr_2_; 99.999%; Sigma-Aldrich), cesium bromide (CsBr; 99.999%; Sigma-Aldrich), anisole (99.7%; Sigma-Aldrich), *N*,*N*-dimethylformamide (DMF; 99.8%; Sigma-Aldrich), dimethyl sulfoxide (DMSO; ≥99.9%; Sigma-Aldrich), and poly(methyl methacrylate) (PMMA; average *M*_w_ ~ 120,000 by gel permeation chromatograph (GPC), Sigma-Aldrich).

### Sample preparation

The typical procedure for preparation was generally consistent with previous work (*32*). Specifically, quartz glass substrates (Corning; dimensions, 2.0 cm by 2.0 cm) were used in the study. These substrates were thoroughly cleaned using Seife Hellmanex III (2%, 50°C) solution, acetone (20°C), and isopropyl alcohol (20°C) for 20 min. Afterward, they underwent further cleaning using an oxygen plasma (Diener Zepto, 50 W, 13.56 MHz, 10 min). The solutions and films were prepared in a N_2_-filled glovebox. The perovskite solution was prepared by mixing CsI (0.06 M), MAI (0.264 M), FAI (0.876 M), PbBr_2_ (0.264 M), and PbI_2_ (0.936 M). The mixture was stirred in a DMF:DMSO (3:1 volume ratio) solvent at 75°C until it was fully dissolved. PMMA (~0.06 mg/ml) was added to the solution, which was then filtered through a PTFE filter (0.45 μm) before use. The precursor solution was spin coated onto the substrates at 2000 rpm for 30 s (acceleration time of 3 s) and at 6000 rpm for 40 s (acceleration time of 5 s). The films were then treated with ~280 μl of anisole, which was dropped onto the film for 25 s before the end of the process. The films were lastly annealed at 100°C for 20 min.

### PL measurement

Spectrally and tr-PL measurements were conducted using a gated CCD setup that comprised a pulsed UV-solid-state laser (dye laser, 343-nm wavelength, 100-Hz repetition rate), a spectrometer (SPEX 270 M from Horiba Jobin Yvon), and an intensified CCD camera (iStar DH720 from Andor Solis). The minimum time resolution of the setup is 1.9 ns. The applied excitation fluence, initial carrier concentration, and initial ∆*E*_F_ were ~1.79 μJ/cm^2^, 5.61 × 10^16^ cm^−3^, and 1.44 eV, respectively. The laser spot size is 3.07 mm in diameter, and the laser beam profile is Gaussian. Additional information about the gated CCD setup can be found in (*32*). Steady-state PL measurements were performed using a LuQY Pro setup (LP20-32, QYB Quantum Yield Berlin GmbH), which can provide the $\Delta E_{F}$ results using the measured quantum yield $Q_{e}^{\text{lum}}$ under different light intensities by $qV_{\text{oc}}^{\text{rad}}-\Delta E_{F}=-k_{B}T\text{ln}\left(Q_{e}^{\text{lum}}\right)$. To obtain the $V_{\text{oc}}^{\text{rad}}$, an external quantum efficiency (EQE) spectrum of a reference device with the same perovskite absorber is needed. Voltage-dependent PL measurements are carried out using a custom-made setup (*31*, *76*) comprising a power supply, a Keithley 2400 SMU, a bias light source, and a CCD camera. The perovskite solar cells, with an active area of 3.0 mm by 3.0 mm, were illuminated with blue LED light (470 nm) of an intensity of 1 sun. During the measurement process, the CCD camera recorded the PL intensity at various voltages along with *J*-*V* curves. Initially, the background was measured, followed by a flat-field correction to obtain the corrected PL intensity. The *J*-*V* curves were measured in both forward and reverse directions, with a voltage step of 0.02 V. The absorption coefficient α of the perovskite film sample being used to simulate the transient PL spectra requires a high dynamic range around the bandgap. Thus, we derived the shape from steady-state PL measurement using Würfel’s generalized Planck’s law in combination with UV-visible measurement to determine the absolute value (*77*, *78*). The absorption coefficient at the excitation wavelength (λ = 343 nm) is determined from spectroscopic ellipsometry, which is performed from the film side using a rotating analyzer ellipsometer (FLS-860, J.A. Woollam Co.) at different incident angles (55°, 60°, and 65°). Gaussian and parameterized semiconductor (PSemi) oscillator function models are used to fit the data and extract the extinction coefficient *k* for the calculation of absorption coefficient using $\alpha =\frac{4\pi k}{\lambda }$.

### Numerical simulation

Simulations were performed using self-developed MATLAB scripts. Three models have been used in total and introduced in Table 1. More detailed description is shown in section S5.

**Table 1.** Models used in this work. NA, not applicable.

Model	Scenario	Relevant effects	Assumption of defect	Figures involved
0D model	Steady-state PL; transient PL	Photogeneration, trapping, detrapping, and recombination	Shallow traps dominated	Fig. 3A and figs. S5 and S13
1D model	PL redshift; transient PL	Photogeneration, trapping, detrapping, recombination, and diffusion	Deep traps dominated	Fig. 1 and figs. S19 and S20
			Shallow traps dominated	Figs. 3 (B to D) and 4 and figs. S5, S8 to S12, S22, and S23
Device model	Device performance	Photogeneration, trapping, detrapping, recombination, and transport (drift and diffusion)	NA	Fig. 5

## Supplementary Materials

### This PDF file includes:

Supplementary Text Figs. S1 to S23 Tables S1 to S3 Sections S1 to S5 References
