# Supplementary material for: Deriving mobility-lifetime products in halide perovskite films from spectrally and time-resolved photoluminescence
Source: Sci Adv. 2025 Apr 16;11(16):eadt1171. doi: 10.1126/sciadv.adt1171 (PMC12002118; doi:10.1126/sciadv.adt1171)
Supplement: Supplementary file 1 — Supplementary Text Figs. S1 to S23 Tables S1 to S3 Sections S1 to S5 References [file sciadv.adt1171_sm.pdf]

Science Advances
AAAS

Supplementary Materials for

Deriving mobility-lifetime products in halide perovskite films from spectrally and time-resolved photoluminescence

Ye Yuan *et al.*

Corresponding author: Genghua Yan, ge.yan@fz-juelich.de; Thomas Kirchartz, t.kirchartz@fz-juelich.de

Sci. Adv. **11**, eadt1171 (2025)
DOI: 10.1126/sciadv.adt1171

This PDF file includes:

Supplementary Text
Sections S1 to S5
Figs. S1 to S23
Tables S1 to S3
References

Supplementary Text

Section 1.

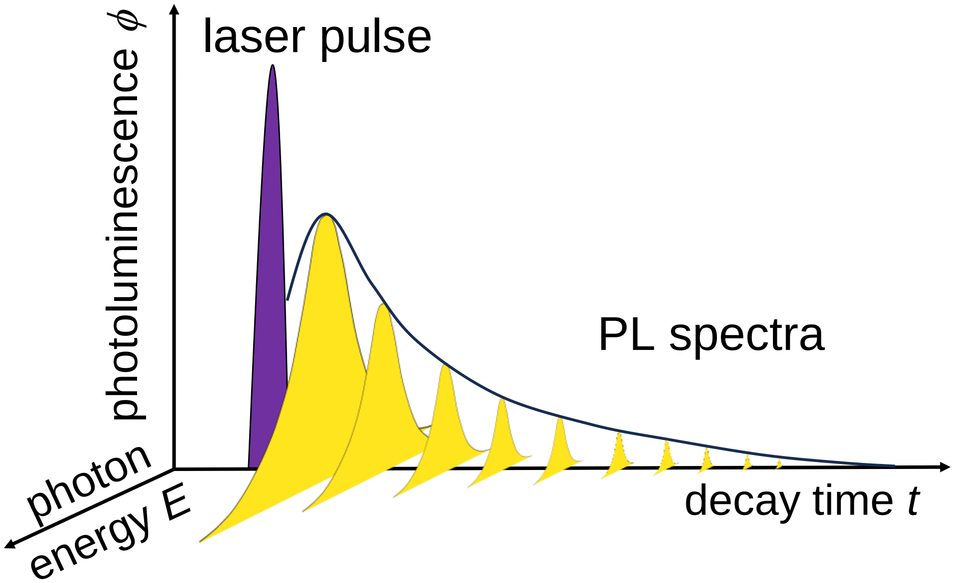

Fig. S1. Schematic illustration of the transient PL measurement using gated CCD technique. A series of time-dependent photoluminescence spectra was recorded during the measurements, whereby each spectrum corresponded to one delay time after excitation. By integrating these spectra, we obtained the photoluminescence intensity at each time point and subsequently obtained a time-dependent PL decay curve. The decay curve primarily provides information about carrier recombination, whereas the time-dependent photoluminescence spectra contain information about carrier diffusion.

Based on the spectral redshift caused by reabsorption, we can determine the out-of-plane mobility of the absorber, which does not require contact or charge transport layers. The acquired data are not sensitive to in-plane diffusion because they do not change the average distance of the charge carrier from the emitting front surface of the film where photons are collected. Another advantage is that this approach does not require strong assumptions on the validity of a certain model (e.g. the space-charge-limited-current method).

Photon recycling can also accelerate the shift of the PL spectra, potentially leading the overestimation of the electronic transport parameters μ and D. This is because photon recycling on average speed up the homogenization process of carriers.(*67*) However, its impact depends on the radiative recombination coefficient and carrier concentration. In our case, the radiative recombination coefficient is approximately 5×10^{-11} cm^3/s (calculated via $k_{\mathrm{rad}} \times n_{\mathrm{i}}^2 \exp(\Delta E_{\mathrm{F}}/k_{\mathrm{B}}\mathrm{T}) = G$), and the experimental data used for fitting are from the first 20 ns (Fig. 3C). The radiative lifetime $\tau_{\mathrm{rad}}=(k_{\mathrm{rad}}\times n)^{-1}$ is shorter than 20 ns only if the carrier concentration $n>1\times10^{18}$ cm^{-3}. However, the initial carrier concentration in our experiment is only 5.6×10^{16} cm^{-3}. Under such conditions, carrier diffusion plays a more significant role in carrier transport than photon recycling.(*67*)

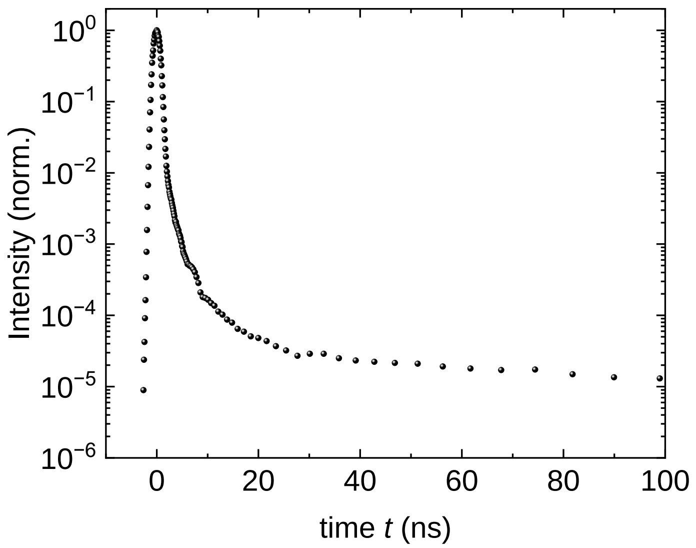

Fig. S2 The instrument response function (IRF) of the gated CCD setup, of which FWHM~1.4 ns.

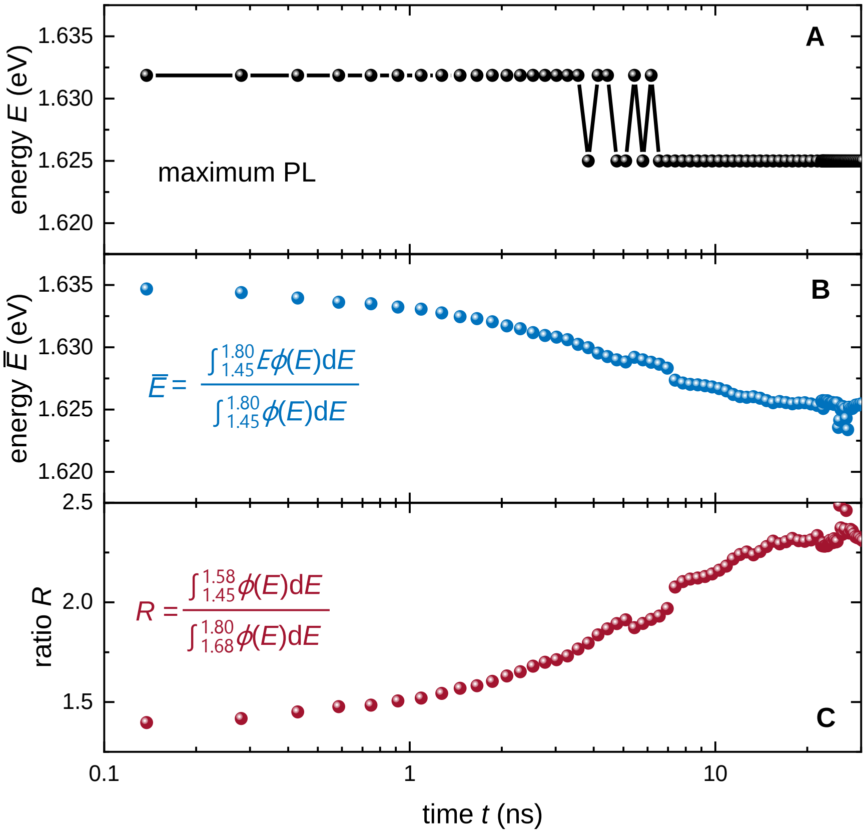

Fig. S3 Quantifying the spectral shift as a function of time. (A) PL peak vs. time. (B) Calculated center of mass of PL spectra vs. time. (C) Calculated ratio of the low-energy region to high-energy region of the PL spectra vs. time. The dataset is the same as the curves shown in Fig. 2, with an illumination intensity of 1.79 μJ/cm^2. In fig. S3A, the energy of the peak position was acquired. In fig. S3B and C, we calculated the center of mass of the PL spectra and the ratio of the low-energy region to the high-energy region, respectively, based on the equations shown in the figure. The results demonstrate that the variation in the center of mass contains the same information as that of the ratio, whereas the change in the peak is not appropriate for the current data owing to the minimal magnitude of the change.

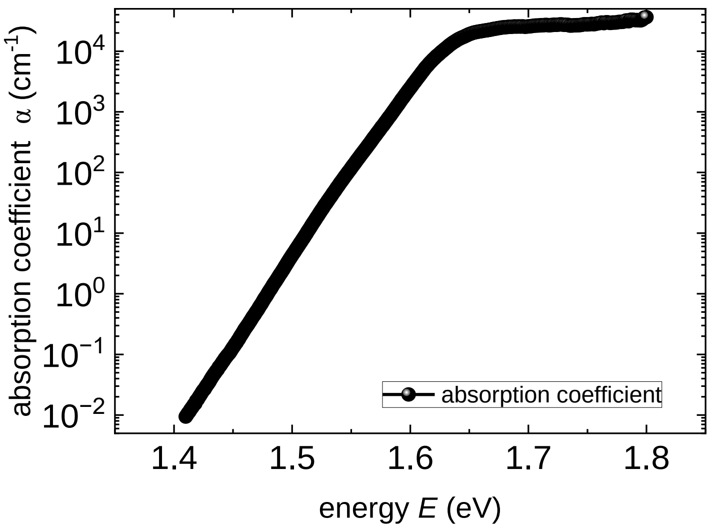

Fig. S4 The calculated absorption coefficient α of perovskite film sample from steady-state photoluminescence measurement in combination with UV-vis measurement. This spectrum is used for the simulation in the 1OD model.

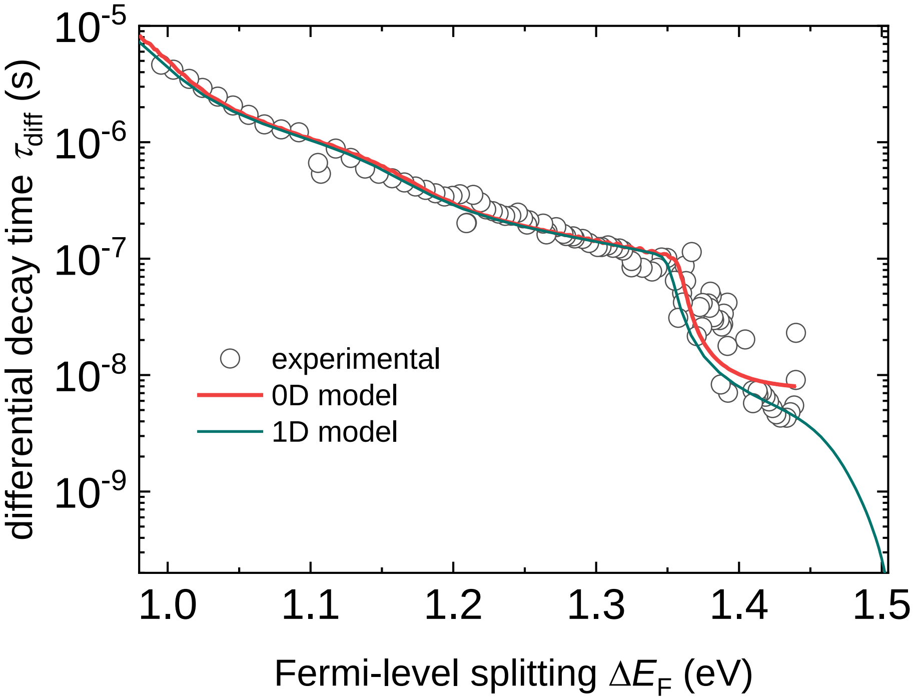

Fig. S5 Comparison of the fitted curves using 0D and 1D model. The results are highly consistent and differ only in the PDE part, which is attributed to the fact that the 0D model does not account for the carrier diffusion process.

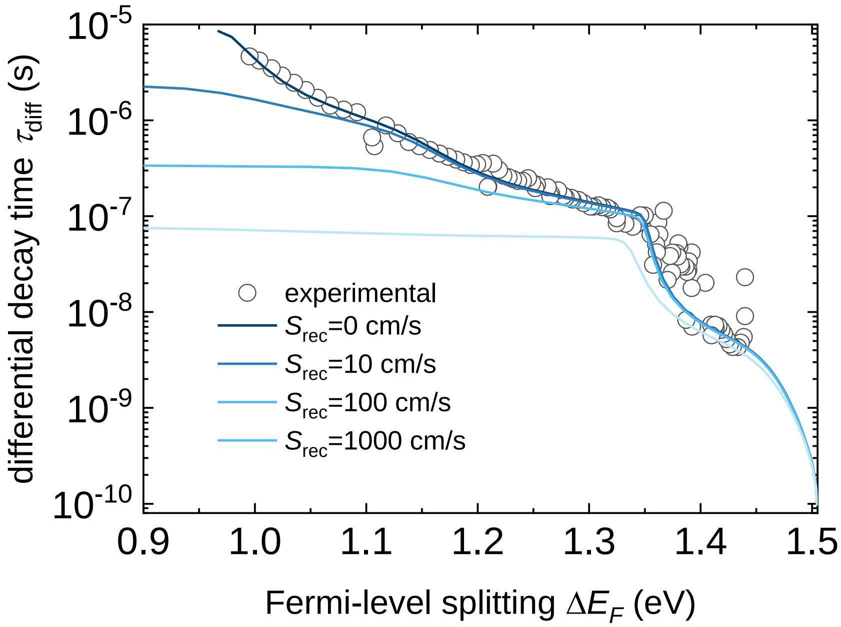

Fig. S6 The influence of surface recombination velocity S_{rec} on τ_{diff} based on simulations assuming a deep interfacial defect.

We propose that the recombination process in our perovskite film is primarily governed by shallow defects, with minimal contribution from deep defects, as supported by tr-PL data.(*32*) In fig. S6, we illustrate the situation that deep defects were at the interface between absorber and either charge transport layer. When the S_{rec} is nonzero, a plateau appears in the τ_{diff} versus ΔE_F plot, and τ_{diff} decreases with increasing S_{rec}. For S_{rec} > 1000 cm/s, τ_{diff} drops below 10^{-7} s, which deviates from our experimental data.

Table S1 Values of parameters related to numerical simulation which can fit the experimental data well (as shown in Fig.3 in main text). All trap-related parameters need to be understood as parameters of bulk traps. We judge that it would be difficult to disentangle shallow bulk from shallow surface traps in this experiment and therefore limit ourselves to shallow bulk traps. For simplicity, acceptor-like traps close to the conduction band were assumed; thus, the defect levels were relative to the top of the valence band. Similar results could be obtained by assuming donor-like traps close to the valence band.

	Parameter	Value	Method
Basic	Bandgap E_g (eV)	1.63	EQE inflection point
	Film thickness d (nm)	550	step profiler
	Radiative recombination coefficient k_{rad} (cm^3/s)	5×10^{-11}	calculation
	Intrinsic carrier concentration n_i ($1/cm^3$)	4.2×10^{4}	calculation
	Initial carrier concentration $n(t=0)$ ($1/cm^3$)	5.6×10^{16}	calculation
	Initial Fermi-level splitting $E_F(t=0)$ (eV)	1.44	calculation
	Absorption coefficient at 343 nm α (1/cm)	4×10^{5}	ellipsometry
	mobility μ (cm^2/Vs)	2	simulation
	diffusion coefficient D (cm^2/s)	0.052	simulation
Defect 1	Defect level E_T (eV)	1.57	simulation
	Defect density N_T ($1/cm^3$)	1.17×10^{18}	simulation
	Electron capture coefficient β_n (cm^3/s)	1.34×10^{-10}	simulation
	Hole capture coefficient β_p (cm^3/s)	7.87×10^{-11}	simulation
	Electron emission coefficient e_n (1/s)	2.52×10^{7}	calculation
	Hole emission coefficient e_p (1/s)	7.42×10^{-19}	calculation
	SRH lifetime# τ_{SRH} (s)	1.72×10^{-8}	calculation
Defect 2	Defect level E_T (eV)	1.53	simulation
	Defect density N_T ($1/cm^3$)	9.58×10^{17}	simulation
	Electron capture coefficient β_n (cm^3/s)	3.03×10^{-11}	simulation
	Hole capture coefficient β_p (cm^3/s)	3.11×10^{-10}	simulation
	Electron emission coefficient e_n (1/s)	1.38×10^{6}	calculation
	Hole emission coefficient e_p (1/s)	1.20×10^{-17}	calculation
	SRH lifetime# τ_{SRH} (s)	3.78×10^{-8}	calculation
Defect 3	Defect level E_T (eV)	1.41	simulation
	Defect density N_T ($1/cm^3$)	1.20×10^{18}	simulation
	Electron capture coefficient β_n (cm^3/s)	4.83×10^{-11}	simulation
	Hole capture coefficient β_p (cm^3/s)	3.05×10^{-10}	simulation
	Electron emission coefficient e_n (1/s)	2.21×10^{4}	calculation
	Hole emission coefficient e_p (1/s)	1.18×10^{-15}	calculation
	SRH lifetime# τ_{SRH} (s)	2.00×10^{-8}	calculation

The SRH lifetime for each trap is calculated based on $\tau_{\mathrm{SRH}} = \tau_{\mathrm{n,SRH}} + \tau_{\mathrm{p,SRH}} = 1/\beta_n N_t + 1/\beta_p N_t$

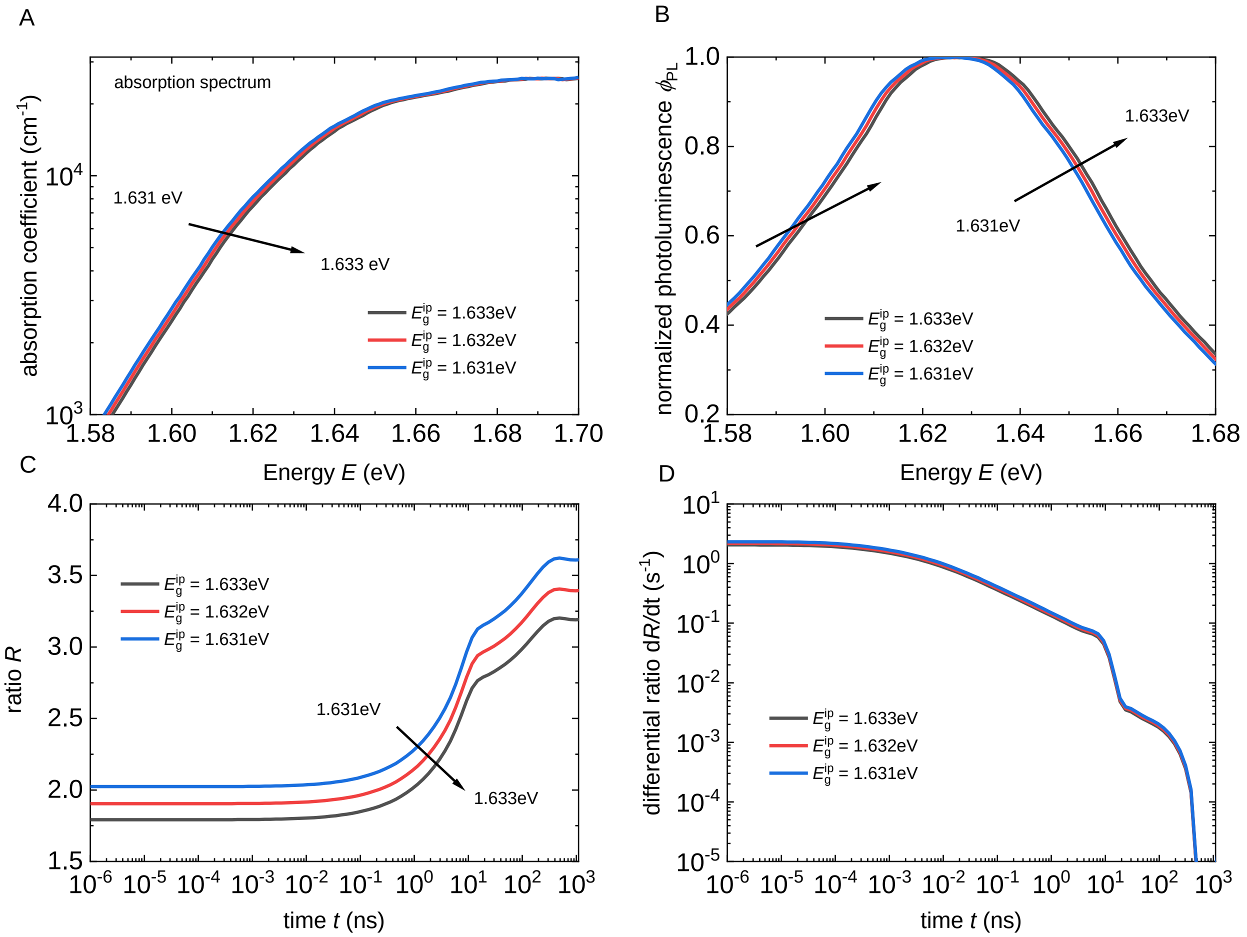

Fig. S7 Simulated results by varying the absorption spectra of perovskite films artificially. (A) absorption spectra. (B) normalized photoluminescence spectra. (C) ratio vs. time. (D) dR/dt vs. time. In the figure, a subtle adjustment is made to the absorption spectrum of the perovskite. Consequently, a slight variation is observed in the simulated photoluminescence spectrum. It is noteworthy that the change in the ratio is highly evident in this instance.

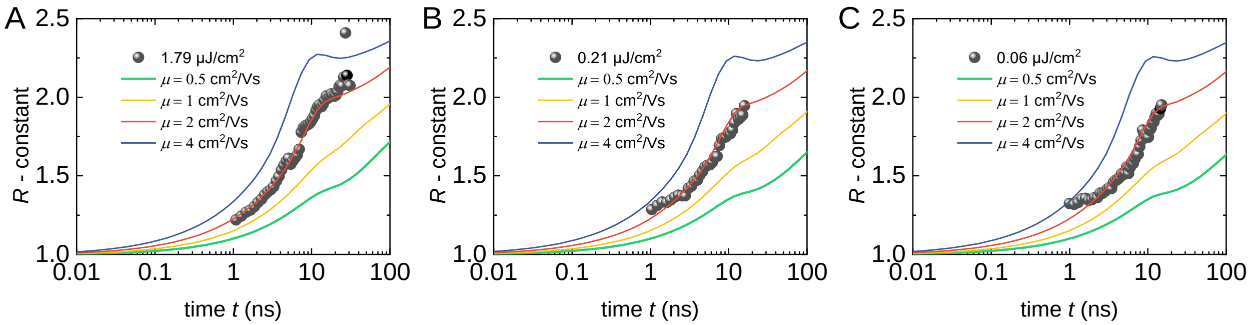

Fig. S8 Experimental ratio vs. time and the corresponding simulated curves under illumination intensity of (A) 1.79 μJ/cm^2, (B) 0.21 μJ/cm^2, (C) 0.06 μJ/cm^2. We skip the data points before 1ns as the minimum gate width of setup is 1.9 ns. The sample shows similar mobility of ~ 2 cm^2/Vs under three illumination intensities.

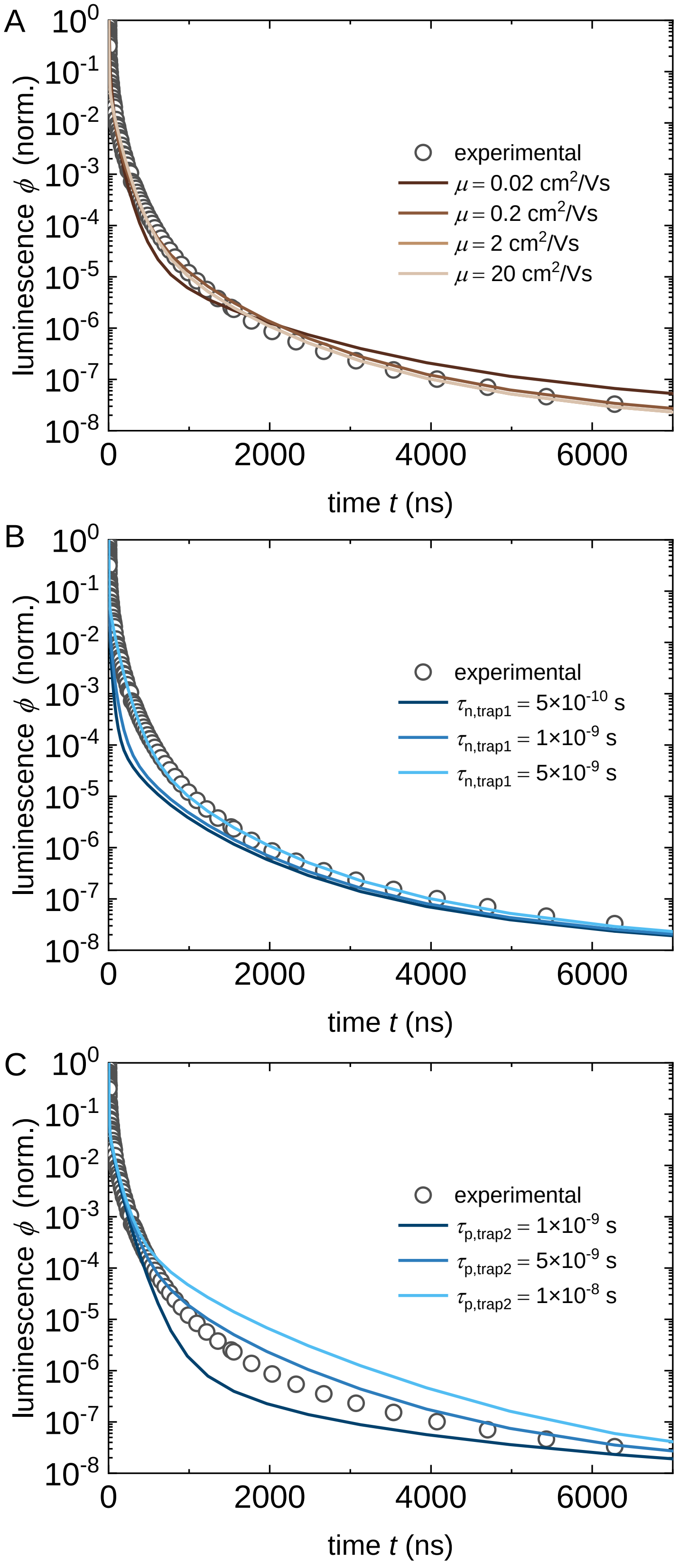

Fig. S9 Influence of (A) carrier mobility, (B) electron lifetime of Trap1 (the shallowest one) and (C) hole lifetime of Trap 2 (the middle one) on the PL decays. The results correspond to Fig. 4 in the main text.

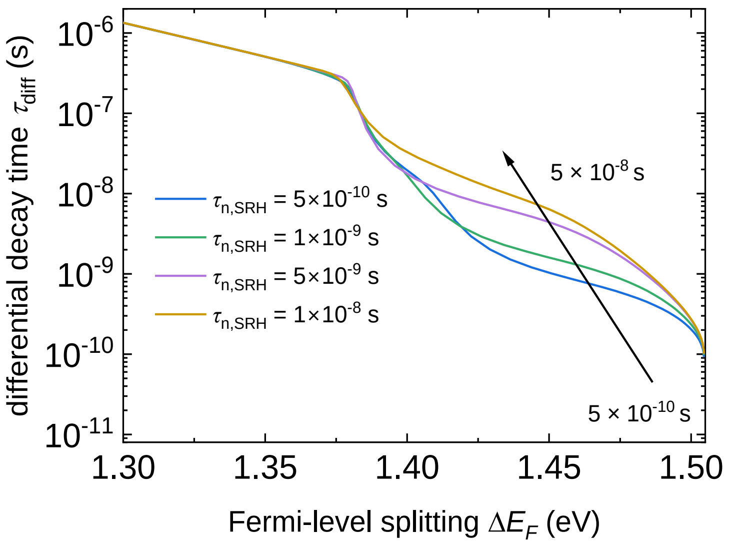

Fig. S10 The influence of SRH lifetime of electrons $\tau_{n,SRH}$ on PL decay. The curves are simulated with only one shallow defect (shallowest one in the table S1) and low radiative recombination coefficient (k_{rad}=1×10^{-13}cm^3/s). The SRH lifetime of electron is defined as $\tau_{n,SRH} = 1/(N_t\beta_n)$.

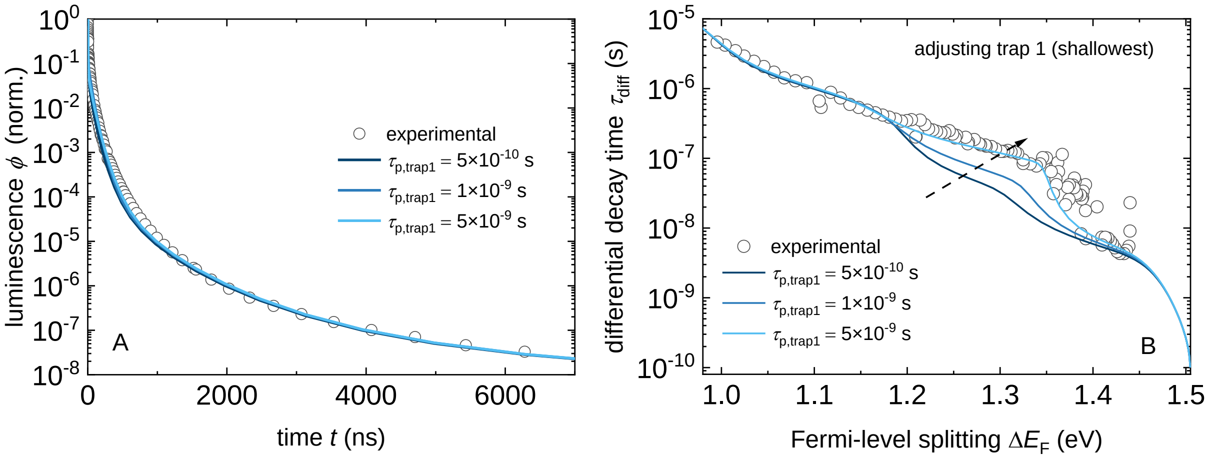

Fig. S11 The influence of hole lifetime of Trap1 (shallowest) on (A) the photoluminescence decay and (B) differential decay time based on the simulation. The figure shows hole lifetime shallowest trap will dominate the earlier times and high fermi-level splitting region. Longer hole lifetime will give a higher decay time.

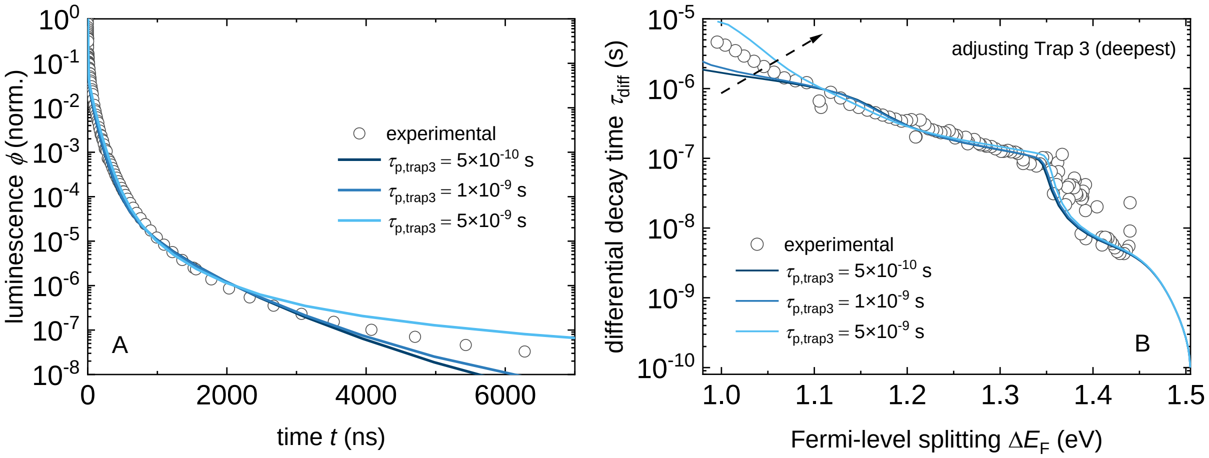

Fig. S12 Influence of hole lifetime of Trap3 (deepest) on (A) photoluminescence decay and (B) differential decay time based on simulation. The figure shows that the hole lifetime of the deepest trap dominates the later time and low Fermi-level splitting region. A longer hole lifetime results in a higher decay time. Together with fig. S11 and Fig. 4E (in the main text), we observe that the covered region shifts from high to low Fermi-level splitting when the trap level changes from shallow to deep.

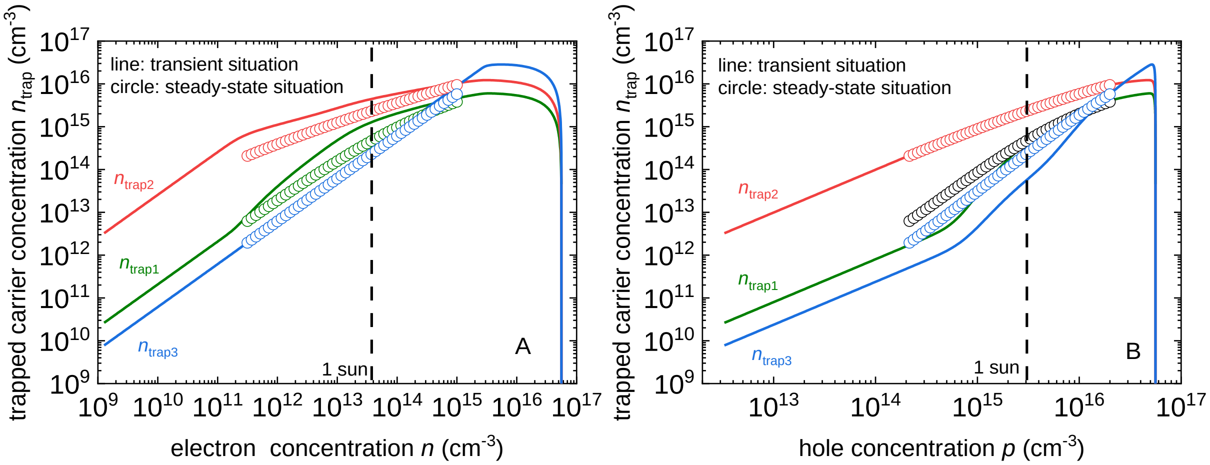

Fig. S13 The trapped carrier concentration in transient and steady-state situations as a function of (A) electron concentration and (B) hole concentration. The simulations were performed using the 0D model, assuming acceptor-like shallow traps close to the conduction band. The parameters of the traps can be found in table S1. The results show that the trapped carrier concentrations are similar in the two situations, with the difference being smaller than one order of magnitude.

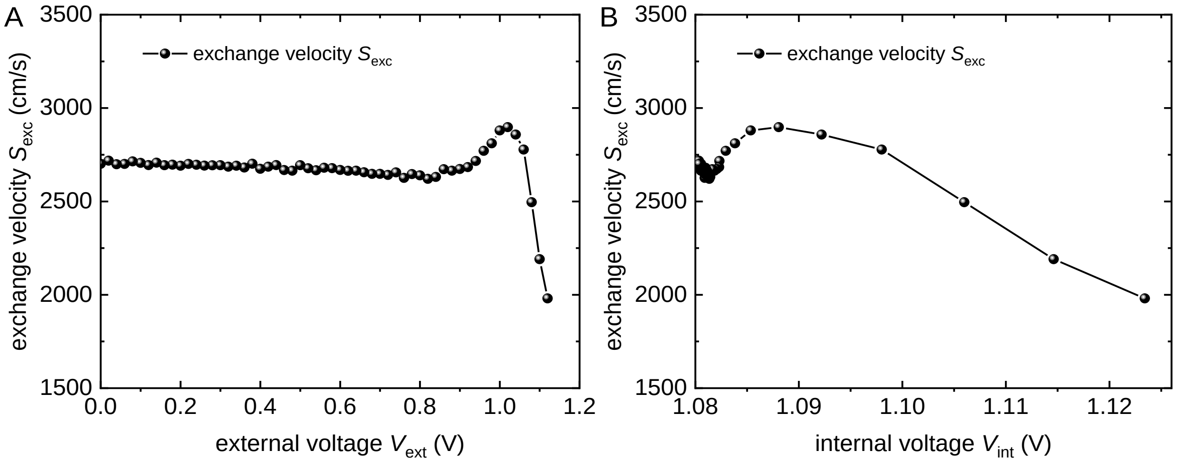

Fig. S14 Exchange velocity S_{exc} along with (A) external and (B) internal voltages, acquiring from voltage-dependent photoluminescence measurement under 1 sun illumination.

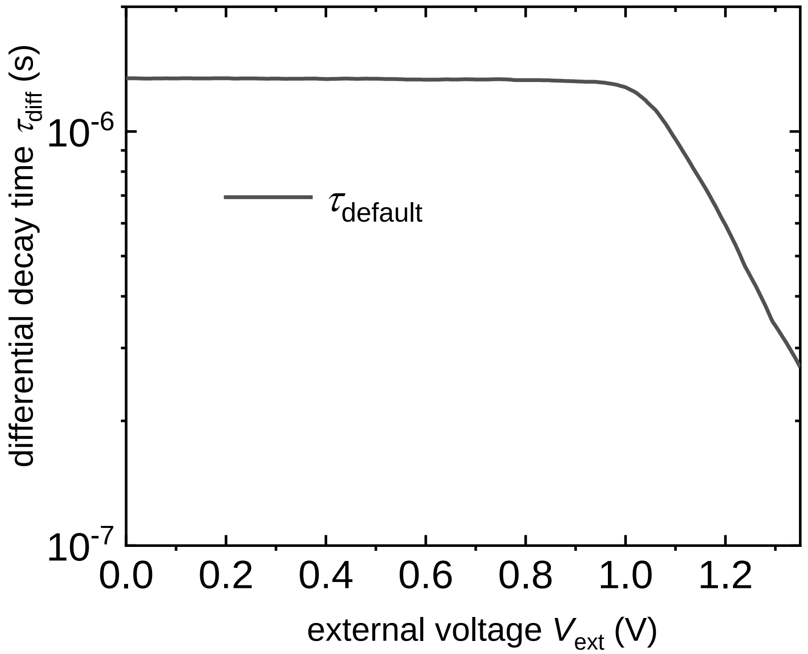

Fig. S15 Differential decay time τ_{diff} versus external voltage V_{ext}. The data of this curve is converted from the data of ΔE_F versus τ_{diff} (Fig. 3B) and V_{ext} versus ΔE_F (Fig. 3E) with the use of interpolation. These data are used as default lifetime for the simulation in Fig 5.

Table S2 The default settings for the calculation in Fig. 5 if not declared otherwise.

Band gap E_g (eV)	1.63
thickness d (nm)	500
mobility μ (cm^2/Vs)	2
Diffusion length L_D(μm)	2.55
Exchange velocity (cm/s)	2700
Short-circuit current J_{sc} (mA/cm^2)	20
Generation rate G ($cm^{-3}\cdot s^{-1}$)	2.5×10^{21}

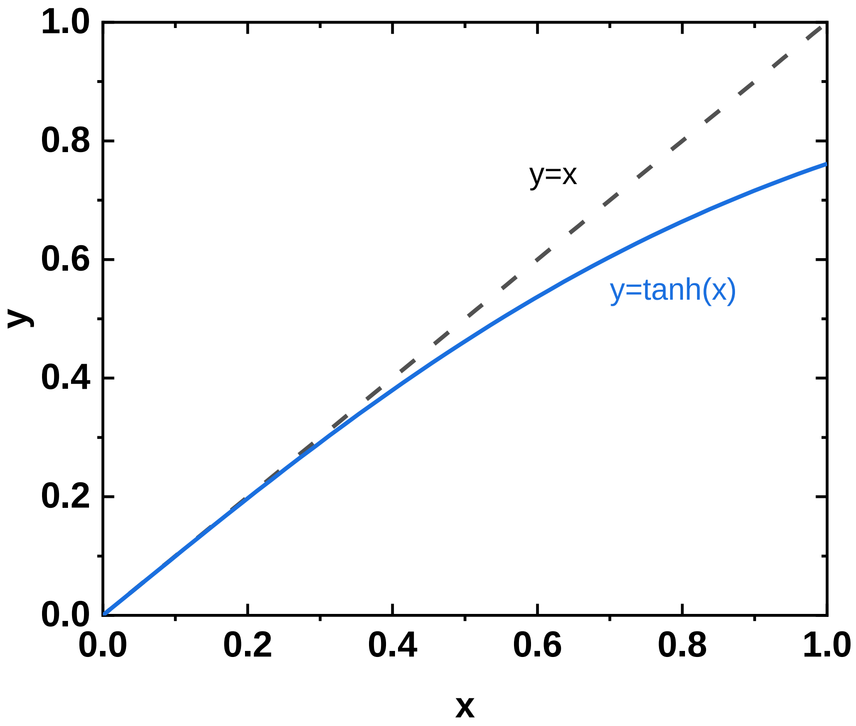

Fig. S16 The curve of hyperbolic tangent function in the region of $0 \le x \le 1$.

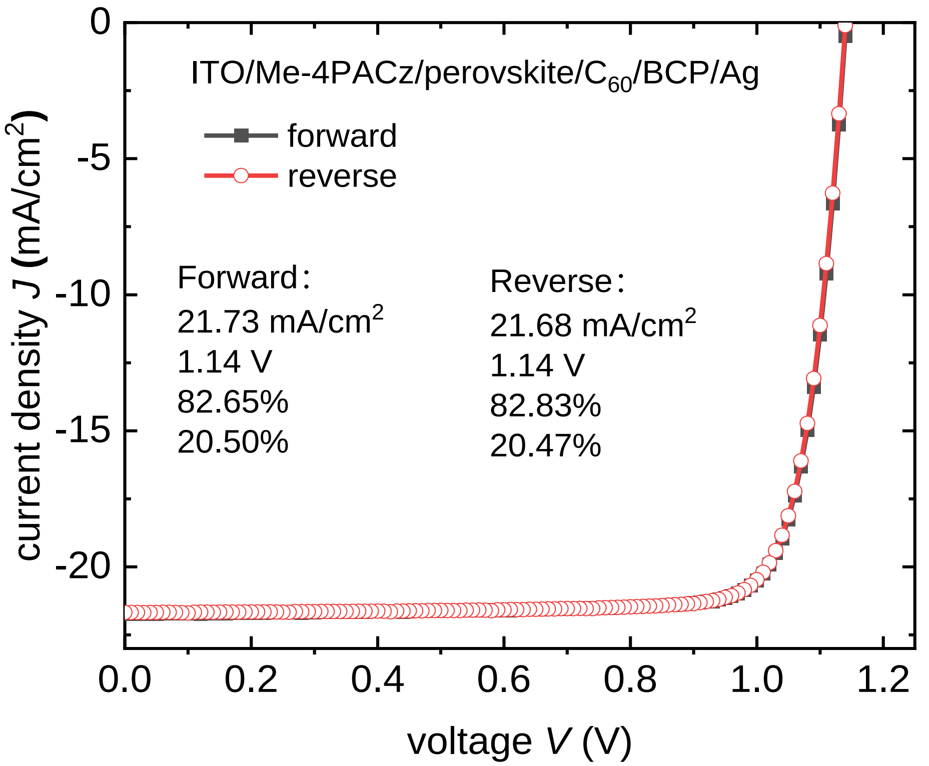

Fig. S17 *JV* curves of ITO/Me-4PACz/perovskite/C_{60}/BCP/Ag solar cells under both forward and reverse scans. The active area is 0.16cm^2. The $Cs_{0.05}FA_{0.73}MA_{0.22}PbI_{2.56}Br_{0.44}$ perovskite films used in this work can achieve an efficiency over 20%.

Section 2 Spectral shifts in a deep-trap dominated model.

In this section, we only consider the deep traps present in the film (as illustrated in fig. S18A). The model accounts for one deep trap in the bulk (identified as "τ_{bulk}") and two deep traps on the surfaces (referred to as "S_L" and "S_R"). Such a model is similar to that of traditional semiconductors, such as Si, GaAs, and CIGS, where carrier recombination is dominated by SRH recombination via deep traps rather than shallow traps. In addition, since shallow traps are not included, the role of each parameter on the spectral shift is clearer. We also use this model for the schematic diagram shown in Fig.1 (main text); thus, this section can be used as a supplement for Fig.1 (main text). We employ the "ratio" to quantify the extent of spectral shift. Following illumination, the carriers are nonuniformly distributed throughout the film. At each location, the carrier undergoes radiative recombination, resulting in photoluminescence. The photoluminescence travels through the film before exiting and is subsequently reabsorbed. The reabsorption length L_{p} is equivalent to the distance from the left surface of the film to the location. Given the inhomogeneous distribution of carriers, we calculate the average reabsorption length, which is defined as,

$$L_{\text{p,ave}} = \frac{\int_0^d L_p(x) n(x) dx}{\int_0^d n(x) dx} = \frac{\int_0^d x \cdot n(x) dx}{\int_0^d n(x) dx} \tag{S1}$$

$L_{\text{p,ave}}$ influences the ratio values and the degree of spectral shifts over time. In fig. S18B, we outline four stages of carrier movement, including the initial stage that occurs immediately after the pulse (i and ii), the diffusion stage (iii), and the final stage when the diffusion process is complete, resulting in a flat distribution of carriers (iv). During the initial stage, the low and high absorption coefficients α are compared. The results illustrate that light penetrates deeper into the film with a lower α, leading to the generation of more photocarriers in the deeper region. A larger value of $L_{\text{p,ave}}$ increases the initial ratio value at $t \approx 0$. Therefore, to achieve a more noticeable change in the ratio, it is preferable to use a higher α value in the experiment. In the second stage, the photocarriers diffuse towards the opposite side of the film, resulting in a continuous increase in the ratio. The rate of change in the ratio is determined by the sample mobility, with a higher mobility leading to a higher diffusion rate. Finally, in the third stage, the carriers are uniformly distributed throughout the film. The $L_{\text{p,ave}}$ value will be approximately equal to half the film thickness (stage iv).

The diagrams depicted in fig. S18 clearly illustrate the results shown in fig. S19. Specifically, an increase in the thickness leads to a higher plateau ratio, whereas a higher absorption coefficient results in a lower initial ratio. Furthermore, a higher mobility reduces the time required to reach the plateau. Additionally, a higher surface-recombination velocity on the left-hand side decreases the carrier density on that side, even when the main diffusion process ends (as depicted in stage (iv)), resulting in a higher plateau ratio ($L_{\text{p,ave}} > d/2$). Conversely, a higher surface-recombination velocity on the right-hand side decreases the plateau ratio. Although the radiative recombination coefficient k_{rad} and deep trap lifetime τ_{bulk} have some influence on the ratio, their impact is limited due to the fact that they do not substantially affect the carrier density distribution, despite dominating carrier density values.

Fig. S19 indicates that only the mobility and absorptance substantially affect the rate of change of the ratio. For the films with similar properties, the absorptance remains constant. By differentiating the ratio with respect to time, we can quantitatively or qualitatively assess the mobility of the films. In fig. S20, the mobility values vary over a broader range, and the simulation parameters are identical to those shown in fig. S19H, except for the adjusted absorption coefficient, which was set to 4×10^5 cm^{-1}, corresponding to the real measured value (based on ellipsometry) of our sample at a UV wavelength (343 nm). Fig. S20B displays the diff. ratio values. It is evident that high mobility is associated with high diff. ratio values over an extended period (approx. 1 fs $\leqslant$ t $\leqslant$ 10 ns). This

provides a basis for qualitatively comparing the mobilities of different films. At a sufficiently early time, the diff. ratio is proportional to mobility, that is, dR/dt = constant×μ. Fig. S20C plots the quantitative relationship between the diff. ratio and mobility at 1 fs, 1 ps, and 1 ns, respectively Notably, at 1 fs, the diff. ratio and mobility fits the equation dR/dt = constant×μ, where the constant ≈1. At 1 ps and 1 ns, they also satisfy exponential relationships. The smaller the time, the smaller the error. It is important to note that the specific relational equations change with film absorptance.

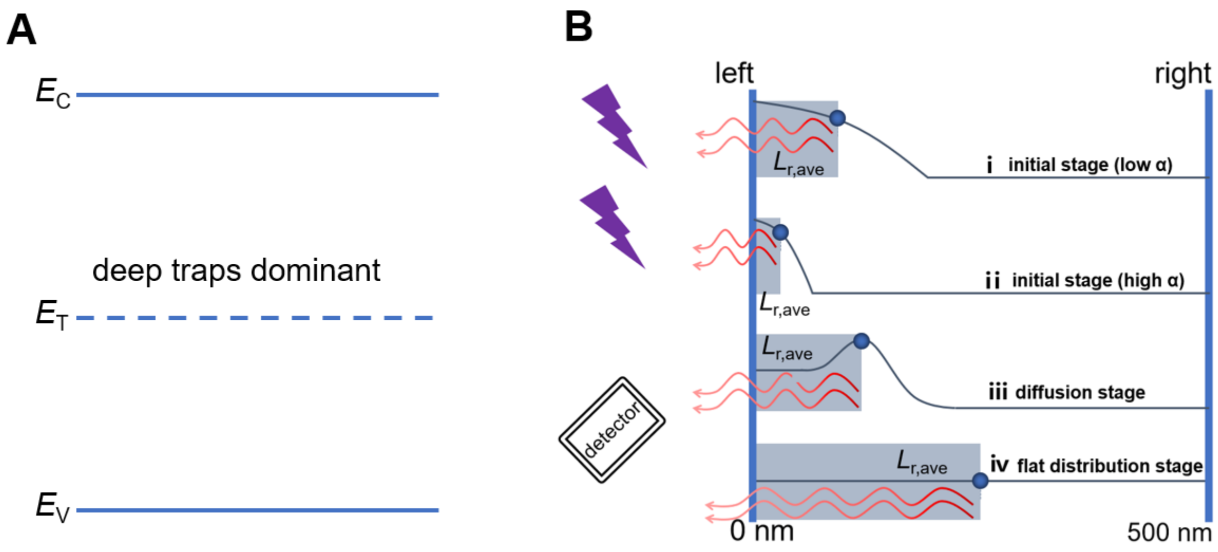

Fig. S18 Schematics of deep traps dominated situation. (A) band diagram (B) carrier movements with 4 stages. The length of shadow region represents $\boldsymbol{L}_{\mathbf{p,ave}}$.

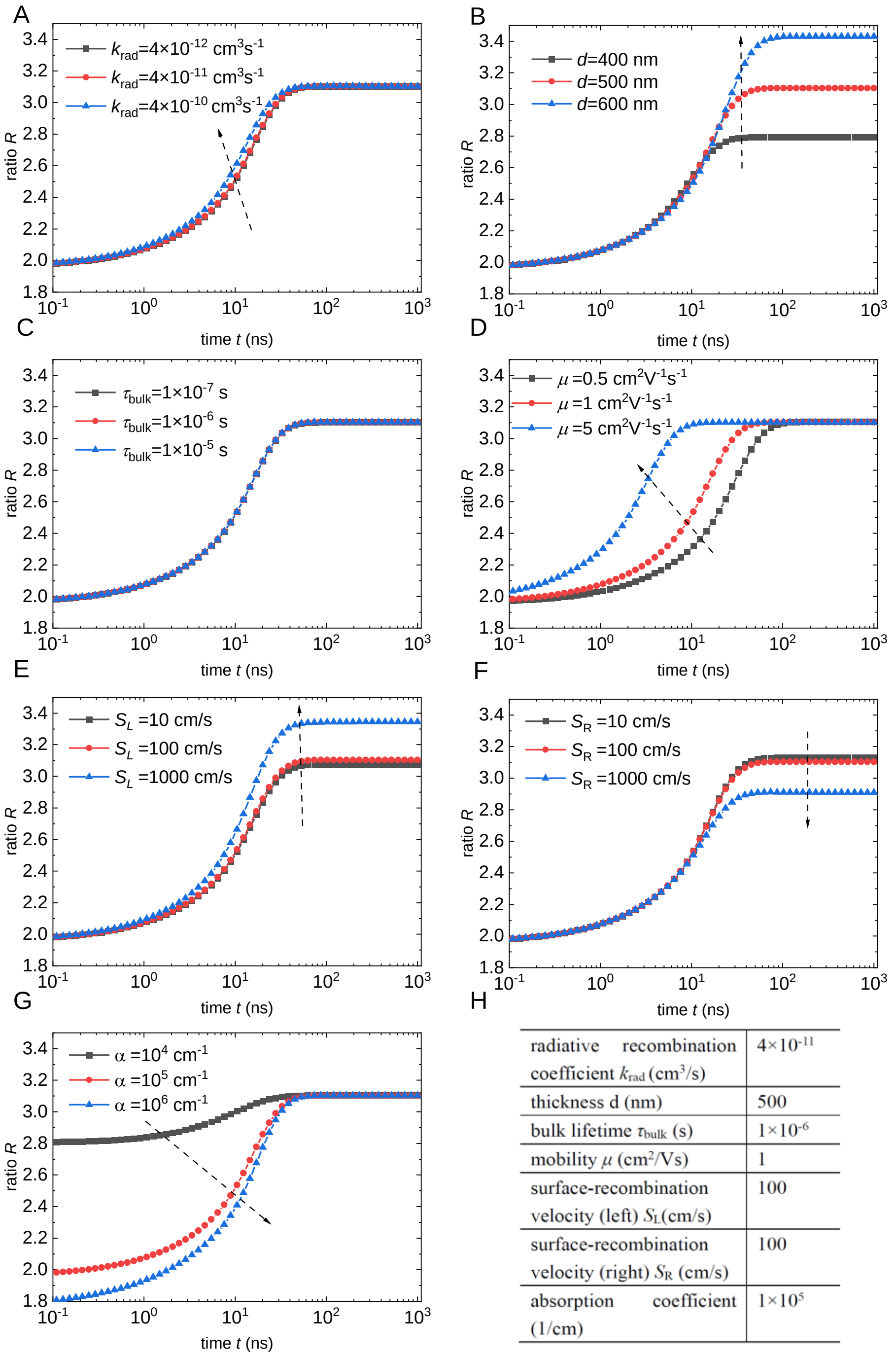

radiative recombination coefficient k_{rad} (cm^3/s)	4×10^{-11}
thickness d (nm)	500
bulk lifetime τ_{bulk} (s)	1×10^{-6}
mobility μ (cm^2/Vs)	1
surface-recombination velocity (left) S_L(cm/s)	100
surface-recombination velocity (right) S_R (cm/s)	100
absorption coefficient (1/cm)	1×10^{5}

Fig. S19 Simulated result for the ratio along with the time. (A) Varying radiative recombination coefficient k_{rad}. (B) Varying film thickness d. (C) Varying deep traps' lifetime in the bulk τ_{bulk}. (D) Varying carrier mobility μ. (E) Varying surface-recombination velocity (left) S_L. (F) Varying surface-recombination velocity (right) S_R. (G) Varying absorption coefficient α. (H) Default parameters in Section 2, if not declared otherwise.

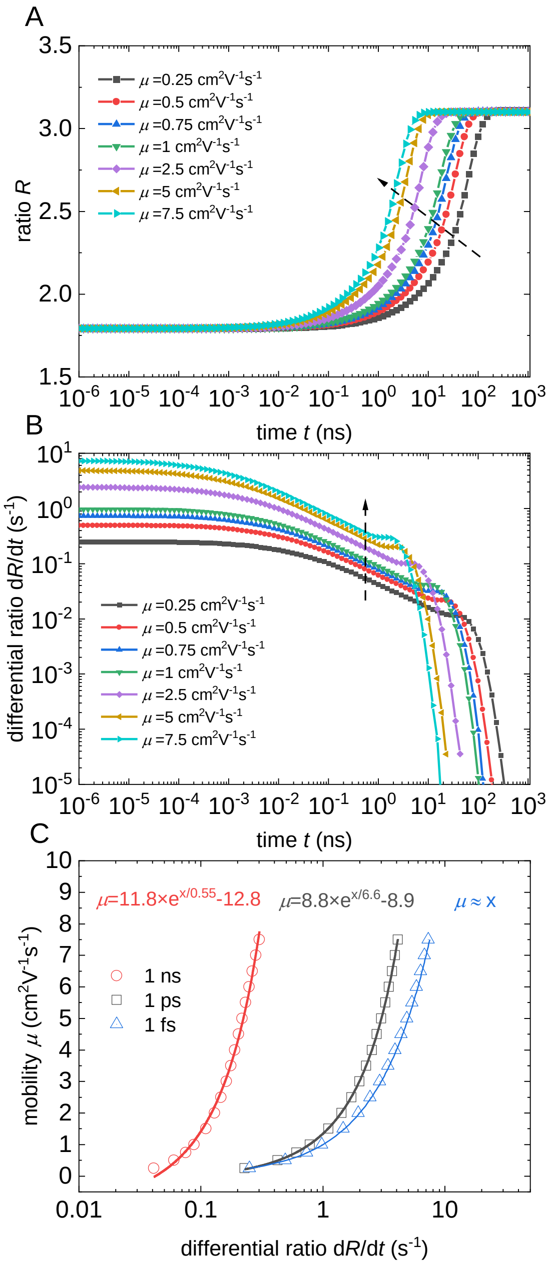

Fig. S20 Simulated result by varying mobility of the films via deep traps dominated model. (A) ratio vs. time. (B) diff. ratio vs. time. (C) mobility vs. diff ratio.

Section 3 Spectral shifts in a shallow-trap dominated model.

An advantage of the 1OD model used in this work is that it can study the influence of shallow traps on the spectral shift. High-quality perovskites exhibit almost no deep traps in the film. The carrier dynamics are primarily influenced by several shallow traps. In this study, we present simulation results depicting three shallow traps, which is consistent with the typical characteristics of our film, as illustrated in ref(*32*). The content in this section is similar to that of real perovskite films, which describe the behavior of shallow traps. In addition, the reason why the experimental data of the ratio R shown in Fig. 2F (main text) did not transform into a fixed constant (as in Fig. 1C) at the long times (i.e. low Fermi-level splitting region) has been explained. The band diagram is shown in fig. S21A. These traps were configured to be acceptor-like, positioned close to the conduction band. The default settings for the simulation are provided in table S3. Unlike in the deep trap scenario, the detrapping process from shallow traps must also be considered. The detrapping process is typically slow and occurs after the diffusion process. Additionally, the left side of the detrapping region occurs before the right side. The processes are detailed in fig. S21B. First, the carriers diffuse from one side to the other to reach an initial flat distribution. During this stage, $L_{\mathrm{p,ave}}$ increases rapidly until it is equals to $d/2$. Subsequently, the carrier density on the left side increases due to detrapping, resulting in a slight decrease in $L_{\mathrm{p,ave}}$. Over time, the detrapping region shifts from left to right, causing $L_{\mathrm{p,ave}}$ to be slightly larger than $d/2$. Moreover, along with the detrapping of the carriers, the diffusion process occurs again owing to the concentration differences. In fig. S22, the impact of shallow traps is demonstrated by varying the properties of Trap 3, which is the deepest among the three shallow traps. Unlike the case of deep traps, the curves in these figures can be divided into three stages that correspond to the three processes shown in fig. S21. In the first stage (Process I), the rapid diffusion of carriers results in a rapid increase in the ratio until it reaches a plateau, reflecting the initial flat distribution of the carriers. In the second stage (Process II), detrapping on the left side becomes the dominant process, causing a slight decrease in the ratio as carriers are released. Finally, in the third stage (Process III), detrapping on the right side leads to an increase in the ratio. It is important to note that there is no clear boundary between these three stages, and the influence of detrapping becomes more pronounced as the trap density and/or electron capture coefficient increases, resulting in more dramatic changes in the ratio (as shown in fig. S22B and S22C). Deeper energy levels and higher hole capture coefficients have little effect on the ratio, as they do not affect the relative distribution of carriers, despite causing more severe recombination (fig. S22A and S22D).

Fig. S23 exhibits the relationship between the differential ratio and mobility in the shallow trap dominated situation. The plateau shapes in this scenario are noticeably distinct from those in the deep trap dominated situation (fig. S20). However, for the first 10 ns, the curves show minimal differences between the two scenarios. This suggests that the detrapping effect of shallow defects is negligible during the initial 10 ns. The differential ratio can still provide a qualitative representation of the mobility situation (fig. S23B). Moreover, the quantitative relationship between the differential ratio and the mobility (fig. S23C) remains consistent at the selected time points when compared to the deep traps situation (fig. S20C).

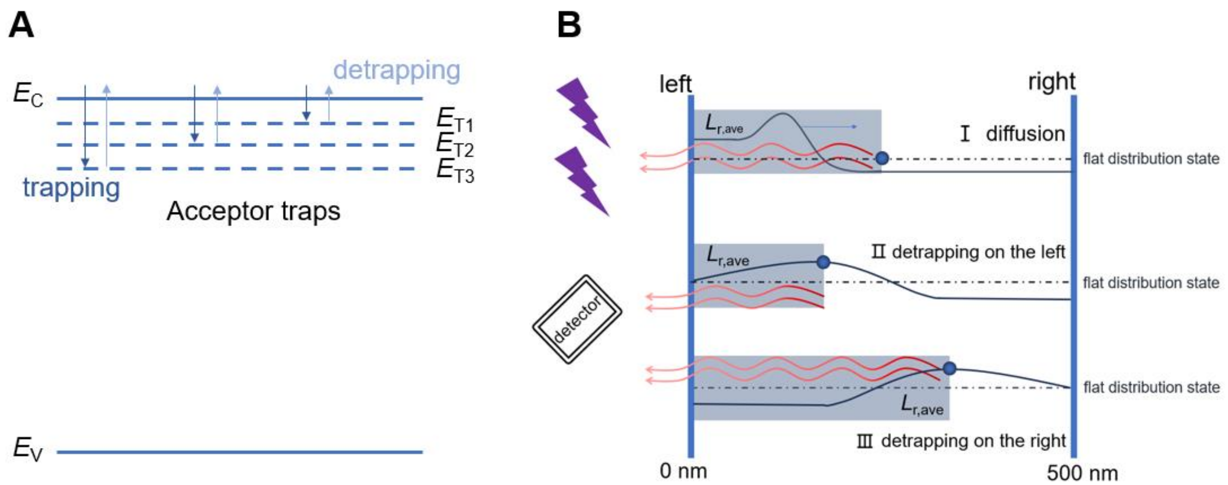

Fig. S21 Schematics of shallow traps dominated situations. (A) band diagram (B) carrier movement schematics with 3 processes. Process (I) shows carrier diffusion from left to right to reach an initial flat distribution state. Process (II) shows the left side of the film release carrier by detrapping after the initial flat distribution state. Process (III) shows the right side of the film release carrier by detrapping after the initial flat distribution state and process (II). The length of shadow region represents $L_{\mathrm{p,ave}}$. The dash dot line represents initial flat distribution state of carrier when main diffusion process is ends.

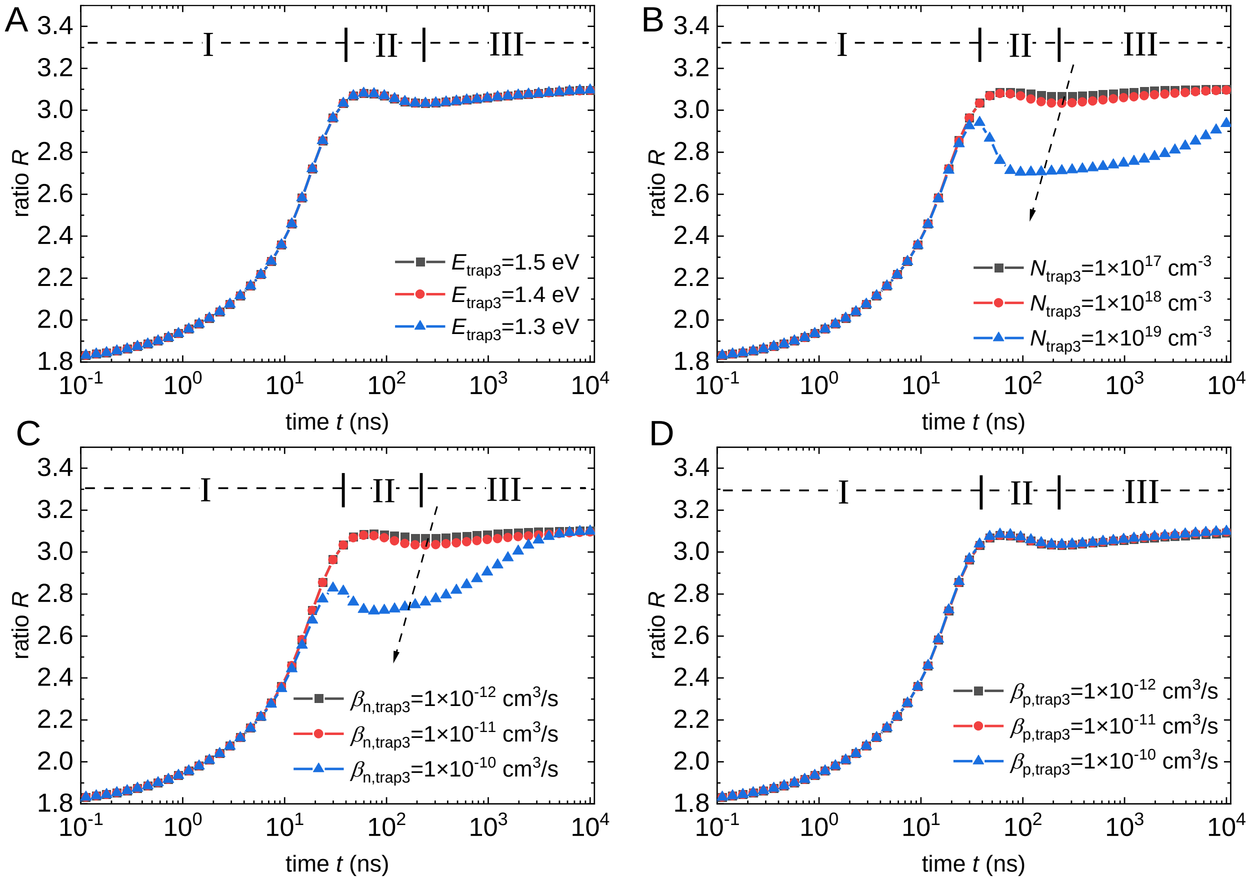

Fig. S22 Simulated result for the ratio along with the time. (A) Varying energy level of trap 3. (B) Varying density of trap 3. (C) Varying electron capture coefficient of trap 3. (D) Varying hole capture coefficient of trap 3.

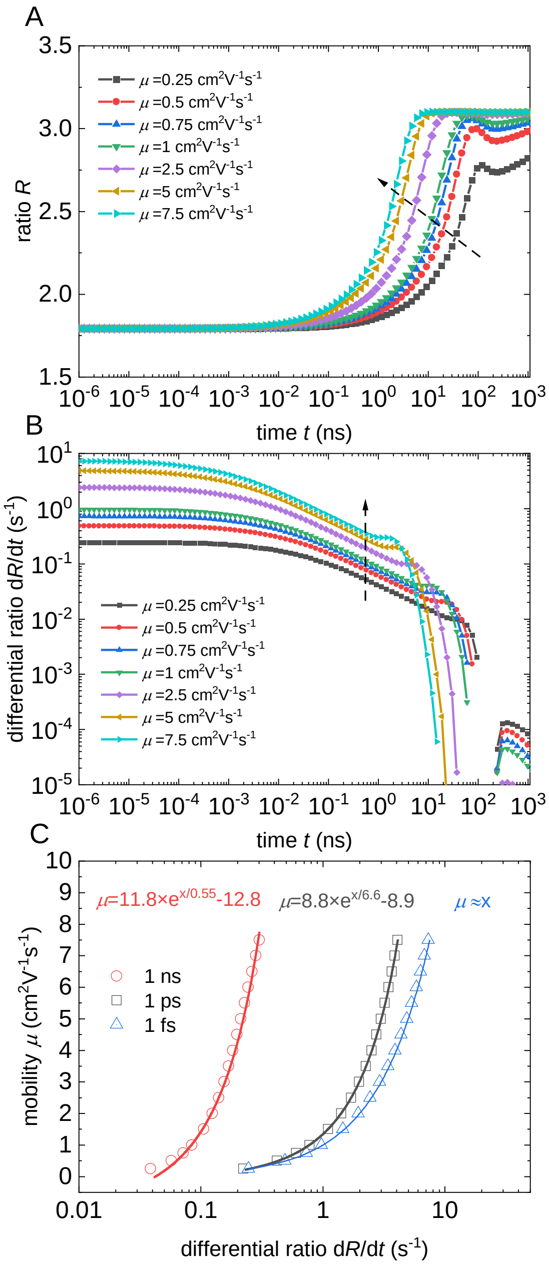

Fig. S23 Simulated result by varying mobility of the films via shallow traps dominated model. (A) ratio vs. time. (B) differential ratio vs. time. (C) mobility vs. differential ratio.

Table S3 The default setting for the simulation for Section 3 if not declared otherwise.

radiative recombination coefficient k_{rad} (cm^3/s)	4×10^{-11}
thickness d (nm)	500
bulk lifetime τ_{bulk} (s)	Infinite
mobility μ (cm^2/Vs)	1
surface-recombination velocity (left) S_L(cm/s)	0
surface-recombination velocity (right) S_R (cm/s)	0
absorption coefficient (1/cm)	4×10^{5}
Trap 1 level E_{trap1} (eV)	1.57
Trap 1 density N_{trap1} (1/cm^3)	1×10^{18}
Electron capture coefficient of Trap 1 $\beta_{n,trap1}$ (cm^3/s)	1×10^{-11}
Hole capture coefficient of Trap 1 $\beta_{p,trap1}$ (cm^3/s)	1×10^{-11}
Trap 2 level E_{trap2} (eV)	1.53
Trap 2 density N_{trap2} (1/cm^3)	1×10^{18}
Electron capture coefficient of Trap 2 $\beta_{n,trap2}$ (cm^3/s)	1×10^{-11}
Hole capture coefficient of Trap 2 $\beta_{p,trap2}$ (cm^3/s)	1×10^{-11}
Trap 3 level E_{trap3} (eV)	4×10^{-11}
Trap 3 density N_{trap3} (1/cm^3)	1×10^{18}
Electron capture coefficient of Trap 3 $\beta_{n,trap3}$ (cm^3/s)	1×10^{-11}
Hole capture coefficient of Trap 3 $\beta_{p,trap3}$ (cm^3/s)	1×10^{-11}

Section 4 Derivation of *J-V* characterization

As demonstrated by Krückemeier et al. (35), in steady state condition, the current density J can be expressed as

$$J = qn_0 S_{\mathrm{exc}} \left[\exp\left(\frac{qV_{\mathrm{ext}}}{2k_{\mathrm{B}}T}\right) - \exp\left(\frac{qV_{\mathrm{int}}}{2k_{\mathrm{B}}T}\right)\right] \tag{S2}$$

where q is the elementary charge, n_0 is the intrinsic carrier concentration, k_B is the Boltzmann constant and T is the temperature, as well as V_{ext} and V_{int} are the external and internal voltage respectively. The quantity S_{exc} is the carrier extraction velocity of the transport layer in units of cm s^{-1}, describing how fast transport of electrons through the ETL and holes through the HTL is, which can be defined by

$$S_{\mathrm{exc}} = \frac{\mu_{\mathrm{CTL}}\, U_{\mathrm{CTL}}/d_{\mathrm{CTL}}}{1 - e^{(-(U_{\mathrm{CTL}}/k_{\mathrm{B}}T))}} \tag{S3}$$

μ_{CTL} is the mobility of the electrons in the ETL and/or of holes in the HTL. $U_{\mathrm{CTL}} = -Fd_{\mathrm{CTL}}$ is the potential difference across the ETL, and d_{CTL} is the CTL thickness.

The transport equation of photogenerated charges (e.g. excess electrons, Δn) under illumination conditions is defined as(*76*)

$$-\frac{L_{\mathrm{D}}^2}{(\tau_{\mathrm{n}} + \tau_{\mathrm{p}})}\frac{d^2\Delta n}{dx^2} + \frac{\Delta n}{(\tau_{\mathrm{n}} + \tau_{\mathrm{p}})} = G \tag{S4}$$

where G is the generation rate of free charges in the absorber, τ_n and τ_p are the bulk lifetimes of the electrons and holes, respectively. By using the assumption of perfect selectivity of contact layers (i.e. the HTL allows only holes to diffuse through it, where $J_n(0)=0$; and the ETL is perfectly selective for electrons, $J_p(d)=0$) as the boundary conditions, the solution of the excess charges Δn in the absorber layer ($0 \le x \le d$) follows

$$\Delta n(x) = G(\tau_{\mathrm{n}} + \tau_{\mathrm{p}})\left[1 - \cosh\left(\frac{x}{L_{\mathrm{D}}}\right) C\right] \tag{S5}$$

where C is a positive integration constant. Afterwards, the current density of excess electrons J_n inside the absorber can be written as

$$J_n(x) = \mu k_B T \frac{d\Delta n}{dx} = -qL_{\mathrm{diff}} G C \sinh\left(\frac{x}{L_{\mathrm{D}}}\right) \tag{S6}$$

By using Equations (S2), (S5) and (S6), as well as the diode equation, where $\Delta n = n_0\left[\exp\left(\frac{qV_{\mathrm{int}}}{2k_BT}\right) - 1\right]$, we obtain the solution for the positive integration constant C as

$$C = \frac{S_{\mathrm{exc}}\left\{G(\tau_n + \tau_p) - n_0\left[\exp\left(\frac{qV_{\mathrm{ext}}}{2k_BT}\right) - 1\right]\right\}}{L_{\mathrm{D}} G \sinh\left(\frac{d}{L_{\mathrm{D}}}\right) + G(\tau_n + \tau_p) S_{\mathrm{exc}} \cosh\left(\frac{d}{L_{\mathrm{D}}}\right)} \tag{S7}$$

Therefore, by substituting Equation (S7) into Equation (S5), and combing with $\Delta n = n_0\left[\exp\left(\frac{qV_{\mathrm{int}}}{2k_BT}\right) - 1\right]$, the current density J as a function of external voltage V_{ext} is obtained as

$$\begin{aligned} J &= qn_0 S_{\mathrm{ext}}\left[\exp\left(\frac{qV_{\mathrm{ext}}}{2k_BT}\right) - 1 - \frac{\Delta n(d)}{n_0}\right] \\ &= qd\left[\frac{\frac{L_{\mathrm{D}}}{d}\tanh\left(\frac{d}{L_{\mathrm{D}}}\right)}{\frac{\mu k_B T}{qL_{\mathrm{D}} S_{\mathrm{exc}}}\tanh\left(\frac{d}{L_{\mathrm{D}}}\right) + 1}\right]\left\{\frac{n_0}{(\tau_n + \tau_p)}\left[\exp\left(\frac{qV_{\mathrm{ext}}}{2k_BT} - 1\right)\right] - G\right\} \end{aligned} \tag{S8}$$

Therefore, if we know the thickness d, mobility μ, diffusion length L_D, lifetime $\tau = \tau_n + \tau_p$ of the absorber layer, as well as the generation rate of free carriers G and the extraction velocity of transport layer S_{exc}, we can reconstruct the *J-V* curves.

Section 5 Numerical models

Two MATLAB scripts, both developed in-house, were employed in this study. The first script (called “0D model”) was utilized to simulate both steady-state (ss-PL) and transient photoluminescence (tr-PL) results, and it relied on coupled rate equations. This logic has been described in previous work.(*32*) With the use of this script, the recombination-related coefficients were obtained.

The second script (called “1D model”) was designed to determine the carrier diffusion coefficient and mobility, besides the recombination coefficients. This script utilized the pdepe function to calculate the electron and hole densities across the depth and time in the film. The specifics of this process are outlined below.

Equation

The behavior of electrons and holes can be described by the continuity equation, which considers both the diffusion and recombination processes. We assume charge neutrality is maintained. Hence there is no electric field and consequently no drift current.

$$\frac{dn}{dt} = -R + D_n \frac{d^2 n}{dx^2} \tag{S9}$$

$$\frac{dp}{dt} = -R + D_p \frac{d^2 p}{dx^2} \tag{S10}$$

The concentration of the trapped electrons is determined by the capture and emission behaviors of the traps. The standard form required for pdepe is,

$$c\left(x,t,u,\frac{\partial u}{\partial x}\right)\frac{\partial u}{\partial t} = x^{-m}\frac{\partial}{\partial x}\left(x^m f\left(x,t,u,\frac{\partial u}{\partial x}\right)\right) + s\left(x,t,u,\frac{\partial u}{\partial x}\right). \tag{S11}$$

The relevant items are.

$$m = 0$$
$$c = [1;1;1;1;1]$$
$$f = [D_\mathrm{n}; D_\mathrm{p}; 0; 0; 0] \times \partial n/\partial x$$

where D_n and D_p are the diffusion coefficients of electrons and holes, respectively. We set them equal in the study i.e., $D_\mathrm{n} = D_\mathrm{p} = \mathrm{D}$.

The “s” term is an array containing five elements, which are used to calculate the electron concentration/hole concentration and the trapped carrier concentration at various points in time. It is important to note that only the recombination processes are considered in this context, as the diffusion process is addressed in the “f” term. The elements of the array are,

$$-(n-n_0)/\tau_\mathrm{deep} - k_\mathrm{rad}(np - n_i^2) - \beta_\mathrm{n}^\mathrm{t1} n(N_\mathrm{t1} - n_\mathrm{t1}) + e_\mathrm{n}^\mathrm{t1} n_\mathrm{t1} - \beta_\mathrm{n}^\mathrm{t2} n(N_\mathrm{t2} - n_\mathrm{t2}) + e_\mathrm{n}^\mathrm{t2} n_\mathrm{t2} - \beta_n^\mathrm{t3} n(N_\mathrm{t3} - n_\mathrm{t3}) + e_\mathrm{n}^\mathrm{t3} n_\mathrm{t3}$$

$$-(p-p_0)/\tau_\mathrm{deep} - k_\mathrm{rad}(np - n_i^2) - \beta_\mathrm{p}^\mathrm{t1} p n_\mathrm{t1} + e_\mathrm{p}^\mathrm{t1}(N_\mathrm{t1} - n_\mathrm{t1}) - \beta_\mathrm{p}^\mathrm{t2} p n_\mathrm{t2} + e_\mathrm{p}^\mathrm{t2}(N_\mathrm{t2} - n_\mathrm{t2}) - \beta_\mathrm{p}^\mathrm{t3} p n_\mathrm{t3} + e_\mathrm{p}^\mathrm{t3}(N_\mathrm{t3} - n_\mathrm{t3})$$

$$\beta_\mathrm{n}^\mathrm{t1} n(N_\mathrm{t1} - n_\mathrm{t1}) - \beta_\mathrm{p}^\mathrm{t1} p n_\mathrm{t1} - e_\mathrm{n}^\mathrm{t1} n_\mathrm{t1} + e_\mathrm{p}^\mathrm{t1}(N_\mathrm{t1} - n_\mathrm{t1})$$

$$\beta_\mathrm{n}^\mathrm{t2} n(N_\mathrm{t2} - n_\mathrm{t2}) - \beta_\mathrm{p}^\mathrm{t2} p n_\mathrm{t2} - e_\mathrm{n}^\mathrm{t2} n_\mathrm{t2} + e_\mathrm{p}^\mathrm{t2}(N_\mathrm{t2} - n_\mathrm{t2})$$

$$\beta_{\mathrm{n}}^{\mathrm{t3}} n(N_{\mathrm{t3}} - n_{\mathrm{t3}}) - \beta_{\mathrm{p}}^{\mathrm{t3}} p n_{\mathrm{t3}} - e_{\mathrm{n}}^{\mathrm{t3}} n_{\mathrm{t3}} + e_{\mathrm{p}}^{\mathrm{t3}}(N_{\mathrm{t3}} - n_{\mathrm{t3}})$$

Initial conditions

Here, we consider that the laser is illuminated on the left side, then the initial condition $u_{(t=0)}$ is,

$$u_{(t=0)} = [n_{(t=0,x=0)} e^{-\alpha \mathrm{x}}; n_{(t=0,x=0)} e^{-\alpha \mathrm{x}}; 0; 0; 0]$$

where $n_{(t=0,x=0)} = \alpha d n_0/(1 - e^{-\alpha d})$. n_0 is the initial carrier concentration, which is determined by the incident laser intensity.

If laser is illuminated on the right side, we have,

$$u_{(t=0)} = [n_{(t=0,x=0)} e^{-\alpha(d-\mathrm{x})}; n_{(t=0,x=0)} e^{-\alpha(d-\mathrm{x})}; 0; 0; 0]$$

Boundary conditions

For electrons and holes, the boundary conditions on the left and right sides are assumed to be,(*76*)(*79*)

$$\frac{j_n}{q} = -D_n \frac{d}{dx} \Delta n|_{x=0} = S_n \Delta n(0) \quad \text{(S12)}$$

$$\frac{j_p}{q} = -D_p \frac{d}{dx} \Delta p|_{x=0} = S_p \Delta p(0) \quad \text{(S13)}$$

$$\frac{j_n}{q} = -D_n \frac{d}{dx} \Delta n|_{x=d} = S_n \Delta n(d) \quad \text{(S14)}$$

$$\frac{j_p}{q} = -D_p \frac{d}{dx} \delta \Delta p|_{x=d} = S_p \Delta p(d) \quad \text{(S15)}$$

The standard form of the boundary conditions in the pdepe function is

$$p(x,t,u) + q(x,t) f\left(x,t,u,\frac{\partial u}{\partial x}\right) = 0 \quad \text{(S16)}$$

So, the boundary conditions at the left side (i.e., x = 0), the term “p” and “q” are,

$$q = [1; 1; 1; 1; 1]$$

$$p = [S_L(n - n_0)/2\,; S_L(p - p_0)/2;\ 0;\ 0;\ 0]$$

where S_L is the recombination velocity of the left side. For the right side of the film (i.e., x = d), it needs to be replaced by the recombination velocity of right side S_{R},

$$p = [S_R(n - n_0)/2\,; S_R(p - p_0)/2;\ 0;\ 0;\ 0]$$

Subsequently, through calculation, we can obtain the carrier profile over time, i.e., $n(x,t)$ and $p(x,t)$.

Calculation for PL spectra, Fermi-level splitting, differential decay time and ratio

We can then obtain the relative PL intensity emitted out the films at various energies and time points using the following equation,

$$\phi_{\mathrm{PL}}(E,t) \propto \int_0^d \left[\alpha(E)\phi_{\mathrm{BB}}(E) e^{-x\alpha(E)} n(x,t) p(x,t)\right] dx \quad \text{(S17)}$$

The reabsorption process is factored in this equation. For comparison, $\phi_{\mathrm{PL}}(E) \propto \int_0^d \alpha(E)\, e^{-x\alpha(E)} dx \phi_{\mathrm{BB}}(E) np = a(E)\phi_{\mathrm{BB}}(E)n$,(*80*) when the reabsorption process is not considered. Integrating over E and then normalizing yields $\phi_{PL}^{normal}(t)$, which represents the

normalized transient PL spectra. To determine the Fermi-level splitting at different time point, the following equation is used,

$$\Delta E_{\mathrm{F}}(t) = \Delta E_{\mathrm{F}}(0) + k_{\mathrm{B}}Tln(\phi_{\mathrm{PL}}^{\mathrm{normal}}(t)/\phi_{\mathrm{PL}}^{\mathrm{normal}}(0))$$

The Fermi-level splitting at t = 0 is denoted as $\Delta E_{\mathrm{F}}(0)$, which can be determined using $\Delta E_{\mathrm{F}}(0) = k_{\mathrm{B}}Tln(\frac{n(0)p(0)}{n_i^2})$. The values of $n(0)$ and $p(0)$are obtained by integrating $n(x,t=0)$ and $p(x,t=0)$ over x. The differential decay time is computed by,

$$\tau_{\mathrm{diff}} = \left(-\frac{1}{2}d\ln(\phi_{\mathrm{PL}})/dt\right)^{-1} \quad \text{(S18)}$$

According to the obtained $\phi_{\mathrm{PL}}(E,t)$, it is possible to integrate separately for the high-energy and low-energy regions and then calculate the ratio between the two. The change in the ratio over time is mainly determined by the mobility.

REFERENCES AND NOTES

1. R. S. Crandall, Modeling of thin-film solar cells: Nonuniform field. *J. Appl. Phys.* **55**, 4418–4425 (1984).

2. R. S. Crandall, Transport in hydrogenated amorphous silicon p-i-n solar cells. *J. Appl. Phys.* **53**, 3350–3352 (1982).

3. T. Kirchartz, J. Bisquert, I. Mora-Sero, G. Garcia-Belmonte, Classification of solar cells according to mechanisms of charge separation and charge collection. *Phys. Chem. Chem. Phys.* **17**, 4007–4014 (2015).

4. D. J. Coutinho, G. C. Faria, D. T. Balogh, R. M. Faria, Influence of charge carriers mobility and lifetime on the performance of bulk heterojunction organic solar cells. *Sol. Energy Mater. Sol. Cells* **143**, 503–509 (2015).

5. J. G. Labram, E. E. Perry, N. R. Venkatesan, M. L. Chabinyc, Steady-state microwave conductivity reveals mobility-lifetime product in methylammonium lead iodide. *Appl. Phys. Lett.* **113**, 153902 (2018).

6. I. Levine, S. Gupta, T. M. Brenner, D. Azulay, O. Millo, G. Hodes, D. Cahen, I. Balberg, Mobility–lifetime products in $MAPbI_3$ films. *J. Phys. Chem. Lett.* **7**, 5219–5226 (2016).

7. J. Zhang, X. Liang, J. Min, J. Zhang, D. Zhang, C. Jin, S. Liang, P. Chen, L. Ling, J. Chen, Y. Shen, L. Wang, Effect of point defects trapping characteristics on mobility-lifetime ($\mu\tau$) product in CdZnTe crystals. *J. Cryst. Growth* **519**, 41–45 (2019).

8. I. Levine, S. Gupta, A. Bera, D. Ceratti, G. Hodes, D. Cahen, D. Guo, T. J. Savenije, J. Ávila, H. J. Bolink, O. Millo, D. Azulay, I. Balberg, Can we use time-resolved measurements to get steady-state transport data for halide perovskites? *J. Appl. Phys.* **124**, 103103 (2018).

9. T. W. Crothers, R. L. Milot, J. B. Patel, E. S. Parrott, J. Schlipf, P. Müller-Buschbaum, M. B. Johnston, L. M. Herz, Photon reabsorption masks intrinsic bimolecular charge-carrier recombination in $CH_3NH_3PbI_3$ perovskite. *Nano Lett.* **17**, 5782–5789 (2017).

10. C. Cho, S. Feldmann, K. M. Yeom, Y.-W. Jang, S. Kahmann, J.-Y. Huang, T. C. J. Yang, M. N. T. Khayyat, Y.-R. Wu, M. Choi, J. H. Noh, S. D. Stranks, N. C. Greenham, Efficient vertical charge transport in polycrystalline halide perovskites revealed by four-dimensional tracking of charge carriers. *Nat. Mater.* **21**, 1388–1395 (2022).

11. S. Chattopadhyay, R. S. Kokenyesi, M. J. Hong, C. L. Watts, J. G. Labram, Resolving in-plane and out-of-plane mobility using time resolved microwave conductivity. *J. Mater. Chem. C* **8**, 10761–10766 (2020).

12. R. Gegevičius, M. Franckevičius, V. Gulbinas, The role of grain boundaries in charge carrier dynamics in polycrystalline metal halide perovskites. *Eur. J. Inorg. Chem.* **2021**, 3519–3527 (2021).

13. D. H. Kim, J. Park, Z. Li, M. Yang, J.-S. Park, I. J. Park, J. Y. Kim, J. J. Berry, G. Rumbles, K. Zhu, 300% Enhancement of carrier mobility in uniaxial-oriented perovskite films formed by topotactic-oriented attachment. *Adv. Mater.* **29**, 10.1002/adma.201606831 (2017).

14. Q. Han, S.-H. Bae, P. Sun, Y.-T. Hsieh, Y. Yang, Y. S. Rim, H. Zhao, Q. Chen, W. Shi, G. Li, Y. Yang, Single crystal formamidinium lead iodide ($FAPbI_3$): Insight into the structural, optical, and electrical properties. *Adv. Mater.* **28**, 2253–2258 (2016).

15. A. A. Zhumekenov, M. I. Saidaminov, M. A. Haque, E. Alarousu, S. P. Sarmah, B. Murali, I. Dursun, X.-H. Miao, A. L. Abdelhady, T. Wu, O. F. Mohammed, O. M. Bakr, Formamidinium lead halide perovskite crystals with unprecedented long carrier dynamics and diffusion length. *ACS Energy Lett.* **1**, 32–37 (2016).

16. W. Rehman, R. L. Milot, G. E. Eperon, C. Wehrenfennig, J. L. Boland, H. J. Snaith, M. B. Johnston, L. M. Herz, Charge-carrier dynamics and mobilities in formamidinium lead mixed-halide perovskites. *Adv. Mater.* **27**, 7938–7944 (2015).

17. G. E. Eperon, S. D. Stranks, C. Menelaou, M. B. Johnston, L. M. Herz, H. J. Snaith, Formamidinium lead trihalide: A broadly tunable perovskite for efficient planar heterojunction solar cells. *Energ. Environ. Sci.* **7**, 982–988 (2014).

18. S. D. Stranks, G. E. Eperon, G. Grancini, C. Menelaou, M. J. P. Alcocer, T. Leijtens, L. M. Herz, A. Petrozza, H. J. Snaith, Electron-hole diffusion lengths exceeding 1 micrometer in an organometal trihalide perovskite absorber. *Science* **342**, 341–344 (2013).

19. G. Xing, N. Mathews, S. Sun, S. S. Lim, Y. M. Lam, M. Gratzel, S. Mhaisalkar, T. C. Sum, Long-range balanced electron- and hole-transport lengths in organic-inorganic $CH_3NH_3PbI_3$. *Science* **342**, 344–347 (2013).

20. R. L. Milot, G. E. Eperon, H. J. Snaith, M. B. Johnston, L. M. Herz, Temperature-dependent charge-carrier dynamics in $CH_3NH_3PbI_3$ perovskite thin films. *Adv. Funct. Mater.* **25**, 6218–6227 (2015).

21. C. S. Ponseca Jr., T. J. Savenije, M. Abdellah, K. Zheng, A. Yartsev, T. Pascher, T. Harlang, P. Chabera, T. Pullerits, A. Stepanov, J.-P. Wolf, V. Sundström, Organometal halide perovskite solar cell materials rationalized: Ultrafast charge generation, high and microsecond-long balanced mobilities, and slow recombination. *J. Am. Chem. Soc.* **136**, 5189–5192 (2014).

22. C. La-O-vorakiat, T. Salim, J. Kadro, M.-T. Khuc, R. Haselsberger, L. Cheng, H. Xia, G. G. Gurzadyan, H. Su, Y. M. Lam, R. A. Marcus, M.-E. Michel-Beyerle, E. E. M. Chia, Elucidating the role of disorder and free-carrier recombination kinetics in $CH_3NH_3PbI_3$ perovskite films. *Nat. Commun.* **6**, 7903 (2015).

23. E. M. Hutter, G. E. Eperon, S. D. Stranks, T. J. Savenije, Charge carriers in planar and meso-structured organic-inorganic perovskites: Mobilities, lifetimes, and concentrations of trap states. *J. Phys. Chem. Lett.* **6**, 3082–3090 (2015).

24. O. G. Reid, M. Yang, N. Kopidakis, K. Zhu, G. Rumbles, Grain-size-limited mobility in methylammonium lead iodide perovskite thin films. *ACS Energy Lett.* **1**, 561–565 (2016).

25. D. A. Valverde-Chávez, C. S. Ponseca, C. C. Stoumpos, A. Yartsev, M. G. Kanatzidis, V. Sundström, D. G. Cooke, Intrinsic femtosecond charge generation dynamics in single crystal $CH_3NH_3PbI_3$. *Energ. Environ. Sci.* **8**, 3700–3707 (2015).

26. O. E. Semonin, G. A. Elbaz, D. B. Straus, T. D. Hull, D. W. Paley, A. M. van der Zande, J. C. Hone, I. Kymissis, C. R. Kagan, X. Roy, J. S. Owen, Limits of carrier diffusion in *n*-type and *p*-type $CH_3NH_3PbI_3$ perovskite single crystals. *J. Phys. Chem. Lett.* **7**, 3510–3518 (2016).

27. Q. Dong, Y. Fang, Y. Shao, P. Mulligan, J. Qiu, L. Cao, J. Huang, Solar cells. Electron-hole diffusion lengths > 175 μm in solution-grown $CH_3NH_3PbI_3$ single crystals. *Science* **347**, 967–970 (2015).

28. M. I. Saidaminov, A. L. Abdelhady, B. Murali, E. Alarousu, V. M. Burlakov, W. Peng, I. Dursun, L. Wang, Y. He, G. Maculan, A. Goriely, T. Wu, O. F. Mohammed, O. M. Bakr, High-quality bulk hybrid perovskite single crystals within minutes by inverse temperature crystallization. *Nat. Commun.* **6**, 7586 (2015).

29. D. Shi, V. Adinolfi, R. Comin, M. Yuan, E. Alarousu, A. Buin, Y. Chen, S. Hoogland, A. Rothenberger, K. Katsiev, Y. Losovyj, X. Zhang, P. A. Dowben, O. F. Mohammed, E. H. Sargent, O. M. Bakr, Low trap-state density and long carrier diffusion in organolead trihalide perovskite single crystals. *Science* **347**, 519–522 (2015).

30. O. Gunawan, S. R. Pae, D. M. Bishop, Y. Virgus, J. H. Noh, N. J. Jeon, Y. S. Lee, X. Shao, T. Todorov, D. B. Mitzi, B. Shin, Carrier-resolved photo-Hall effect. *Nature* **575**, 151–155 (2019).

31. J. Siekmann, A. Kulkarni, S. Akel, B. Klingebiel, M. Saliba, U. Rau, T. Kirchartz, Characterizing the influence of charge extraction layers on the performance of triple-cation perovskite solar cells. *Adv. Energy Mater.* **13**, 2300448 (2023).

32. Y. Yuan, G. Yan, C. Dreessen, T. Rudolph, M. Hülsbeck, B. Klingebiel, J. Ye, U. Rau, T. Kirchartz, Shallow defects and variable photoluminescence decay times up to 280 μs in triple-cation perovskites. *Nat. Mater.* **23**, 391–397 (2024).

33. D. Kiermasch, A. Baumann, M. Fischer, V. Dyakonov, K. Tvingstedt, Revisiting lifetimes from transient electrical characterization of thin film solar cells; a capacitive concern evaluated for silicon, organic and perovskite devices. *Energ. Environ. Sci.* **11**, 629–640 (2018).

34. L. Krückemeier, Z. Liu, B. Krogmeier, U. Rau, T. Kirchartz, Consistent interpretation of electrical and optical transients in halide perovskite layers and solar cells. *Adv. Energy Mater.* **11**, 2102290 (2021).

35. L. Krückemeier, Z. Liu, T. Kirchartz, U. Rau, Quantifying charge extraction and recombination using the rise and decay of the transient photovoltage of perovskite solar cells. *Adv. Mater.* **35**, 2300872 (2023).

36. L. Castaner, E. Vilamajo, J. Llaberia, J. Garrido, Investigations of the OCVD transients in solar cells. *J. Phys. D Appl. Phys.* **14**, 1867–1876 (1981).

37. A. Cuevas, F. Recart, Capacitive effects in quasi-steady-state voltage and lifetime measurements of silicon devices. *J. Appl. Phys.* **98**, 074507 (2005).

38. A. Musiienko, F. Yang, T. W. Gries, C. Frasca, D. Friedrich, A. Al-Ashouri, E. Sağlamkaya, F. Lang, D. Kojda, Y.-T. Huang, V. Stacchini, R. L. Z. Hoye, M. Ahmadi, A. Kanak, A. Abate, Resolving electron and hole transport properties in semiconductor materials by constant light-induced magneto transport. *Nat. Commun.* **15**, 316 (2024).

39. O. Gunawan, C. Kim, B. Nainggolan, M. Lee, J. Shin, D. S. Kim, Y. Jo, M. Kim, J. Euvrard, D. Bishop, F. Libsch, T. K. Todorov, Y. Kim, B. Shin, Electronic trap detection with carrier-resolved photo-Hall effect. arXiv:2411.16092 (2024).

40. M. Schleuning, M. Kölbach, F. F. Abdi, K. Schwarzburg, M. Stolterfoht, R. Eichberger, R. van de Krol, D. Friedrich, H. Hempel, Generalized method to extract carrier diffusion length from photoconductivity transients: Cases of $BiVO_4$, halide perovskites, and amorphous and crystalline silicon. *PRX Energy* **1**, 023008 (2022).

41. J. Ye, M. M. Byranvand, C. O. Martínez, R. L. Z. Hoye, M. Saliba, L. Polavarapu, Defect passivation in lead-halide perovskite nanocrystals and thin films: Toward efficient LEDs and solar cells. *Angew. Chem. Int. Ed. Engl.* **60**, 21636–21660 (2021).

42. A. R. Srimath Kandada, A. Petrozza, Research update: Luminescence in lead halide perovskites. *APL Mater.* **4**, 091506 (2016).

43. V. S. Chirvony, S. González-Carrero, I. Suárez, R. E. Galian, M. Sessolo, H. J. Bolink, J. P. Martínez-Pastor, J. Pérez-Prieto, Delayed luminescence in lead halide perovskite nanocrystals. *J. Phys. Chem. C* **121**, 13381–13390 (2017).

44. D. N. Dirin, L. Protesescu, D. Trummer, I. V. Kochetygov, S. Yakunin, F. Krumeich, N. P. Stadie, M. V. Kovalenko, Harnessing defect-tolerance at the nanoscale: Highly luminescent lead halide perovskite nanocrystals in mesoporous silica matrixes. *Nano Lett.* **16**, 5866–5874 (2016).

45. H. Jin, E. Debroye, M. Keshavarz, I. G. Scheblykin, M. B. J. Roeffaers, J. Hofkens, J. A. Steele, It's a trap! On the nature of localised states and charge trapping in lead halide perovskites. *Mater. Horizons* **7**, 397–410 (2020).

46. F. Deschler, M. Price, S. Pathak, L. E. Klintberg, D.-D. Jarausch, R. Higler, S. Hüttner, T. Leijtens, S. D. Stranks, H. J. Snaith, M. Atatüre, R. T. Phillips, R. H. Friend, High photoluminescence efficiency and optically pumped lasing in solution-processed mixed halide perovskite semiconductors. *J. Phys. Chem. Lett.* **5**, 1421–1426 (2014).

47. R. K. Ahrenkiel, Minority-carrier lifetime in III-V semiconductors. *Semicond. Phys. Appl.* **39**, 39–150 (1993).

48. C. J. Hages, A. Redinger, S. Levcenko, H. Hempel, M. J. Koeper, R. Agrawal, D. Greiner, C. A. Kaufmann, T. Unold, Identifying the real minority carrier lifetime in nonideal semiconductors: A case study of kesterite materials. *Adv. Energy Mater.* **7**, 1700167 (2017).

49. T. Unold, L. Gutay, "Photoluminescence analysis of thin-film solar cells" in *Advanced Characterization Techniques for Thin Film Solar Cells* (Wiley-VCH Verlag GmbH & Co. KGaA, 2011), pp. 151–175.

50. G. D. Gilliland, Photoluminescence spectroscopy of crystalline semiconductors. *Mater. Sci. Eng. R Rep.* **18**, 99–399 (1997).

51. C. Huang, S. Wu, A. M. Sanchez, J. J. P. Peters, R. Beanland, J. S. Ross, P. Rivera, W. Yao, D. H. Cobden, X. Xu, Lateral heterojunctions within monolayer MoSe2–WSe2 semiconductors. *Nat. Mater.* **13**, 1096–1101 (2014).

52. M. A. Reshchikov, Measurement and analysis of photoluminescence in GaN. *J. Appl. Phys.* **129**, 121101 (2021).

53. H. Nan, Z. Wang, W. Wang, Z. Liang, Y. Lu, Q. Chen, D. He, P. Tan, F. Miao, X. Wang, J. Wang, Z. Ni, Strong photoluminescence enhancement of MoS_2 through defect engineering and oxygen bonding. *ACS Nano* **8**, 5738–5745 (2014).

54. X. Zhang, H. Dong, W. Hu, Organic semiconductor single crystals for electronics and photonics. *Adv. Mater.* **30**, 1801048 (2018).

55. H. Yu, X. Cui, X. Xu, W. Yao, Valley excitons in two-dimensional semiconductors. *Natl. Sci. Rev.* **2**, 57–70 (2015).

56. V. Sarritzu, N. Sestu, D. Marongiu, X. Chang, S. Masi, A. Rizzo, S. Colella, F. Quochi, M. Saba, A. Mura, G. Bongiovanni, Optical determination of Shockley-Read-Hall and interface recombination currents in hybrid perovskites. *Sci. Rep.* **7**, 44629 (2017).

57. M. Stolterfoht, C. M. Wolff, J. A. Márquez, S. S. Zhang, C. J. Hages, D. Rothhardt, S. Albrecht, P. L. Burn, P. Meredith, T. Unold, D. Neher, Visualization and suppression of interfacial recombination for high-efficiency large-area pin perovskite solar cells. *Nat. Energy* **3**, 847–854 (2018).

58. S. D. Stranks, V. M. Burlakov, T. Leijtens, J. M. Ball, A. Goriely, H. J. Snaith, Recombination kinetics in organic-inorganic perovskites: Excitons, free charge, and subgap states. *Phys. Rev. Appl.* **2**, 034007 (2014).

59. M. Stolterfoht, V. M. Le Corre, M. Feuerstein, P. Caprioglio, L. J. A. Koster, D. Neher, Voltage-dependent photoluminescence and how it correlates with the fill factor and open-circuit voltage in perovskite solar cells. *ACS Energy Lett.* **4**, 2887–2892 (2019).

60. Y. Guo, O. Yaffe, T. D. Hull, J. S. Owen, D. R. Reichman, L. E. Brus, Dynamic emission Stokes shift and liquid-like dielectric solvation of band edge carriers in lead-halide perovskites. *Nat. Commun.* **10**, 1175 (2019).

61. A. D. Wright, C. Verdi, R. L. Milot, G. E. Eperon, M. A. Pérez-Osorio, H. J. Snaith, F. Giustino, M. B. Johnston, L. M. Herz, Electron–phonon coupling in hybrid lead halide perovskites. *Nat. Commun.* **7**, 10.1038/ncomms11755 (2016).

62. P. Caprioglio, M. Stolterfoht, C. M. Wolff, T. Unold, B. Rech, S. Albrecht, D. Neher, On the relation between the open-circuit voltage and quasi-Fermi level splitting in efficient perovskite solar cells. *Adv. Energy Mater.* **9**, 1901631 (2019).

63. F. Peña-Camargo, J. Thiesbrummel, H. Hempel, A. Musiienko, V. M. Le Corre, J. Diekmann, J. Warby, T. Unold, F. Lang, D. Neher, M. Stolterfoht, Revealing the doping density in perovskite solar cells and its impact on device performance. *Appl. Phys. Rev.* **9**, 021409 (2022).

64. H. Hempel, M. Stolterfoht, O. Karalis, T. Unold, The potential of geminate pairs in lead halide perovskite revealed via time-resolved photoluminescence. arXiv:2409.06382 (2024).

65. P. Würfel, T. Trupke, T. Puzzer, E. Schaffer, W. Warta, S. W. Glunz, Diffusion lengths of silicon solar cells from luminescence images. *J. Appl. Phys.* **101**, 123110 (2007).

66. A. Roigé, J. Alvarez, A. Jaffré, T. Desrues, D. Muñoz, I. Martín, R. Alcubilla, J.-P. Kleider, Effects of photon reabsorption phenomena in confocal micro-photoluminescence measurements in crystalline silicon. *J. Appl. Phys.* **121**, 063101 (2017).

67. F. Staub, I. Anusca, D. C. Lupascu, U. Rau, T. Kirchartz, Effect of reabsorption and photon recycling on photoluminescence spectra and transients in lead-halide perovskite crystals. *J. Phys. Mater.* **3**, 025003 (2020).

68. L. Krückemeier, B. Krogmeier, Z. Liu, U. Rau, T. Kirchartz, Understanding transient photoluminescence in halide perovskite layer stacks and solar cells. *Adv. Energy Mater.* **11**, 2003489 (2021).

69. P. Wurfel, The chemical potential of radiation. *J. Phys. C Solid State Phys.* **15**, 3967–3985 (1982).

70. A. Kiligaridis, P. A. Frantsuzov, A. Yangui, S. Seth, J. Li, Q. An, Y. Vaynzof, I. G. Scheblykin, Are Shockley-Read-Hall and ABC models valid for lead halide perovskites? *Nat. Commun.* **12**, 3329 (2021).

71. C. Cho, Y.-W. Jang, S. Lee, Y. Vaynzof, M. Choi, J. H. Noh, K. Leo, Effects of photon recycling and scattering in high-performance perovskite solar cells. *Sci. Adv.* **7**, eabj1363 (2021).

72. O. Breitenstein, An alternative one-diode model for illuminated solar cells. *Energy Procedia* **55**, 30–37 (2014).

73. U. Rau, V. Huhn, B. E. Pieters, Luminescence analysis of charge-carrier separation and internal series-resistance losses in $Cu(In,Ga)Se_2$ solar cells. *Phys. Rev. Appl.* **14**, 014046 (2020).

74. R. S. Crandall, Modeling of thin film solar cells: Uniform field approximation. *J. Appl. Phys.* **54**, 7176–7186 (1983).

75. O. J. Sandberg, J. Kurpiers, M. Stolterfoht, D. Neher, P. Meredith, S. Shoaee, A. Armin, On the question of the need for a built-in potential in perovskite solar cells. *Adv. Mater. Interfaces* **7**, 2000041 (2020).

76. S. Akel, A. Kulkarni, U. Rau, T. Kirchartz, Relevance of long diffusion lengths for efficient halide perovskite solar cells. *PRX Energy* **2**, 013004 (2023).

77. E. Daub, P. Würfel, Ultralow values of the absorption coefficient of Si obtained from luminescence. *Phys. Rev. Lett.* **74**, 1020–1023 (1995).

78. C. Barugkin, J. Cong, T. Duong, S. Rahman, H. T. Nguyen, D. Macdonald, T. P. White, K. R. Catchpole, Ultralow absorption coefficient and temperature dependence of radiative recombination of $CH_3NH_3PbI_3$ perovskite from photoluminescence. *J. Phys. Chem. Lett.* **6**, 767–772 (2015).

79. K. Misiakos, F. A. Lindholm, Minority-carrier accumulation at the base edge of a junction space-charge region under short-circuit conditions. *Solid State Electron.* **30**, 755–758 (1987).

80. T. Kirchartz, U. Rau, Detailed balance and reciprocity in solar cells. *Phys. Status Solidi (a)* **205**, 2737–2751 (2008).
